# The exceptional finding of *Locus* 2 at Dehesilla Cave and the Middle Neolithic ritual funerary practices of the Iberian Peninsula

**DOI:** 10.1371/journal.pone.0236961

**Published:** 2020-08-13

**Authors:** Daniel García-Rivero, Ruth Taylor, Cláudia Umbelino, T. Douglas Price, Esteban García- Viñas, Eloísa Bernáldez-Sánchez, Guillem Pérez-Jordà, Leonor Peña-Chocarro, María Barrera-Cruz, Juan F. Gibaja-Bao, Manuel J. Díaz-Rodríguez, Patricia Monteiro, Juan C. Vera-Rodríguez, Javier Pérez-González

**Affiliations:** 1 Departamento de Prehistoria y Arqueología, Universidad de Sevilla, Seville, Spain; 2 Research Centre for Anthropology and Health, Department of Life Sciences, Universidade de Coimbra, Coimbra, Portugal; 3 Department of Anthropology, University of Wisconsin-Madison, Madison, WI, United States of America; 4 Instituto Andaluz de Patrimonio Histórico, Junta de Andalucía, Seville, Spain; 5 Centro de Ciencias Humanas y Sociales, CSIC, Madrid, Spain; 6 Institución Milà y Fontanals, CSIC, Barcelona, Spain; 7 Interdisciplinary Center for Archaeology and Evolution of Human Behavior, Universidade do Algarve, Faro, Portugal; 8 Departamento de Historia I, Universidad de Huelva, Huelva, Spain; 9 Wellrounded 360º, Nerja, Spain; University at Buffalo - The State University of New York, UNITED STATES

## Abstract

There is a significant number of funerary contexts for the Early Neolithic in the Iberian Peninsula, and the body of information is much larger for the Late Neolithic. In contrast, the archaeological information available for the period in between (ca. 4800-4400/4200 cal BC) is scarce. This period, generally called Middle Neolithic, is the least well-known of the peninsular Neolithic sequence, and at present there is no specific synthesis on this topic at the peninsular scale. In 2017, an exceptional funerary context was discovered at Dehesilla Cave (Sierra de Cádiz, Southern Iberian Peninsula), providing radiocarbon dates which place it at the beginning of this little-known Middle Neolithic period, specifically between ca. 4800–4550 cal BC. *Locus* 2 is a deposition constituted by two adult human skulls and the skeleton of a very young sheep/goat, associated with stone structures and a hearth, and a number of pots, stone and bone tools and charred plant remains. The objectives of this paper are, firstly, to present the new archaeological context documented at Dehesilla Cave, supported by a wide range of data provided by interdisciplinary methods. The dataset is diverse in nature: stratigraphic, osteological, isotopic, zoological, artifactual, botanical and radiocarbon results are presented together. Secondly, to place this finding within the general context of the contemporaneous sites known in the Iberian Peninsula through a systematic review of the available evidence. This enables not only the formulation of explanations of the singular new context, but also to infer the possible ritual funerary behaviours and practices in the 5^th^ millennium cal BC in the Iberian Peninsula.

## 1. Introduction and objectives

Neolithic funerary practices in the Iberian Peninsula are relatively well known. In the current century, this field has been the object of several regional analyses at different scales [e.g. 1–9], and some supra-regional syntheses [10, 11]. However, the present state of knowledge is unevenly distributed between the different archaeological periods that make up the general framework of the Neolithic.

Currently, there is a large number of sites attributed to the Early Neolithic [12], conventionally dated in the Iberian Peninsula between ca. 5600–4800 cal BC. At the least, 30 sites display some kind of funerary contexts. The funerary evidence known at present for this period is most abundantly from cave sites. Disarticulated or isolated human bones are often found with other artifactual and faunal remains, probably partly due to postdepositional processes. Inhumations are generally individual, although several burials may coexist in the same rooms or common areas of the cavities. There are some cases of secondary burials and rare multiple burials. At the same time, burials are documented at open-air sites, where they tend to belong to individual and very rarely multiple inhumations deposited in pits within settlement contexts. Generally, the bodies are placed in a flexed position, lying on the side, with variable assemblages of grave goods, essentially combinations of pottery, stone tools, bone and/or shell elements, and frequently faunal remains [11].

The body of information available for the funerary practices of the Late Neolithic is much larger. However, the chronological definition of this period in calendar years is more complex, due to the greater degree of regional variation displayed by the archaeological record. In some areas, the onset of the Late Neolithic is attributed to the end of the 5^th^ millenium cal BC, but only becomes generalised throughout the Iberian Peninsula during the course of the 4^th^ millennium cal BC. During this period, burials inside caves are still documented, although funerary practices mostly take place in the open, in graves for individual or multiple burials within settlement areas or, more commonly, in areas designated as burial grounds. The types of funerary structures are diverse. There are several documented open-air necropoli (inhumations in graves), and artificial caves used as burial chambers. The monumental nature of some of the earth and stone burial structures is a distinguishing trait of the Late Neolithic, and the emergence of megalithism is generally linked to this period [11, 13, but see 14, 15].

In contrast, the available archaeological information, especially funerary data, is notably scarcer for the time-span between these two periods. The lower threshold is problematic, this period may be placed approximately between 4800-4400/4200 cal BC. The scarcity and great diversity of the archaeological record at this time has generated a great deal of controversy in the artifactual characterisation, the typological synthesis, and the periodisation of the central part of the 5^th^ millennium cal BC [see for example, 16]. Undoubtedly, this period, generally called Middle Neolithic, is the least well-known of the Neolithic sequence of the Iberian Peninsula, especially with regards to the funerary record that occupies us here, and there is presently no specific synthesis on this subject at the peninsular scale.

In 2017, during the archaeological excavations at Dehesilla Cave (Sierra de Cádiz), a depositional context was discovered, providing radiocarbon dates placing it at the beginning of the little-known Middle Neolithic period, specifically between ca. 4800–4550 cal BC. The find is an exceptional ritual funerary deposition (*Locus* 2) constituted by two adult human skulls and the skeleton of a very young sheep/goat, associated with a stone structure and a hearth, and a number of pots, stone and bone tools and charred plant remains. The finding takes on particular importance, not only because of its singular characteristics but also, considering the background briefly presented above, because it offers a unique opportunity to advance our knowledge about the funerary and ritual practices of the populations of the elusive central span of the 5^th^ millennium cal BC in the Iberian Peninsula.

The main objectives of this paper are therefore, firstly, to present the new depositional context documented at Dehesilla Cave, supported by a wide range of empirical data obtained by interdisciplinary methods; and secondly, to place this find within the general context of the body of contemporaneous funerary data known at present in the Iberian Peninsula. This task implies a comparative approach, which enables not only the formulation of a likely interpretation of the new context presented here but also the systematic review of the current evidence available in the Iberian Peninsula. The specific and comparative information will therefore allow us to highlight the singular characteristics of the *Locus* 2 deposition, and to shed new light on the ritual funerary behaviours in practice during the Middle Neolithic throughout the Iberian Peninsula.

## 2. Data and methods

**Fig 1:**
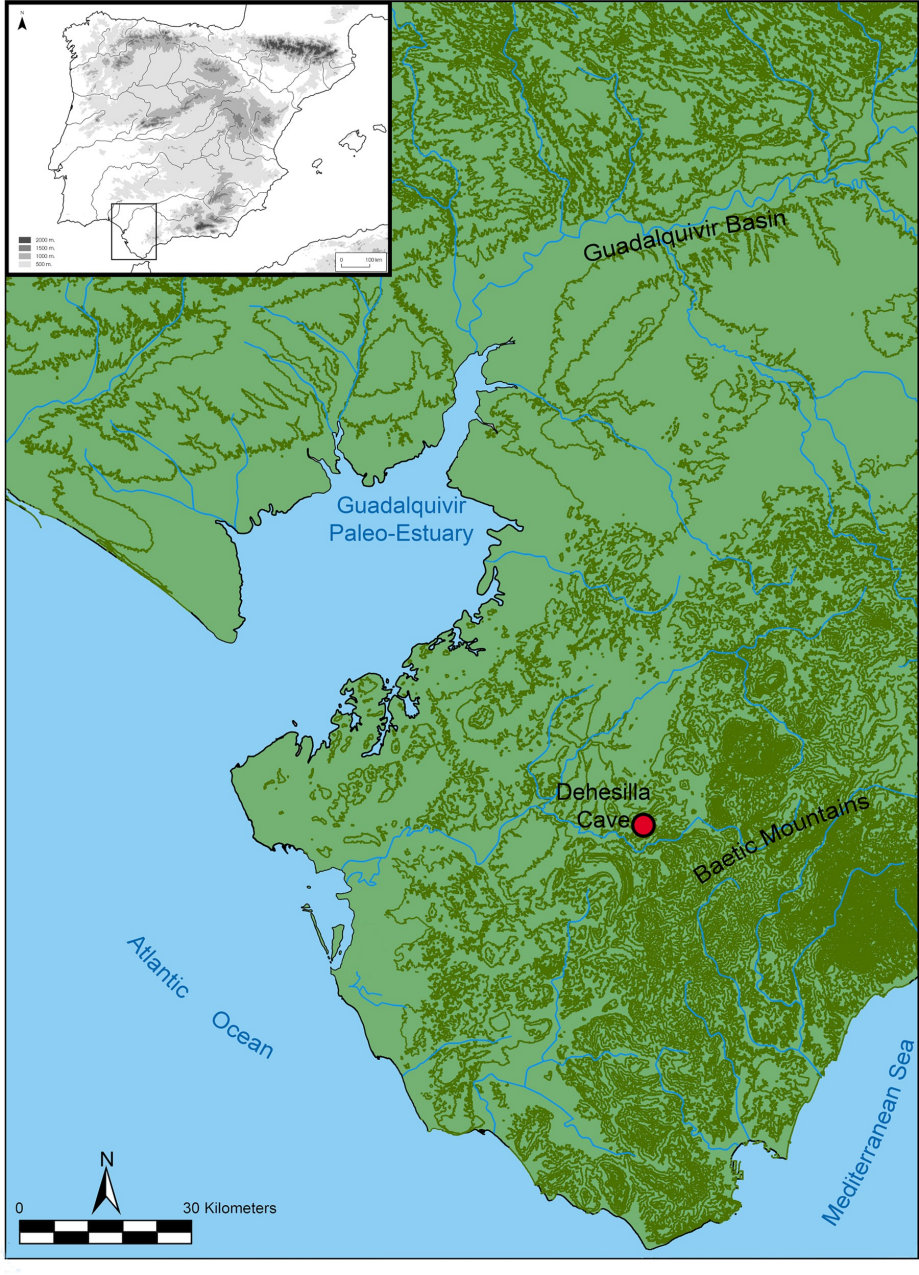
Location of Dehesilla Cave (Theme map bases: [21]).

The data presented in this paper are provided by the recent excavations carried out within the framework of the project “Dehesilla Cave: archaeological and environmental studies for the knowledge of the Prehistoric human occupation of the Sierra de Cádiz”, directed by one of the authors (DGR) (Fig 1). This project conducted a first excavation season in 2016 that led to the identification of several Medieval occupation phases outside the mouth of the cave [17, 18], and a complete Neolithic sequence in the room of the cave nearest the entrance [19] in which previous excavations had been carried out in the 1970s and 80s [20]. The unpublished data presented here come specifically from the second excavation season, in 2017, and from the excavation area identified as C006 located in Room 4, one of the inner-most spaces of Dehesilla Cave.

The data are diverse in nature: stratigraphic, osteological, isotopic, zoological, artifactual, botanical (seeds, fruits and wood) and radiocarbon results are presented here together. The work presented in this article does not raise any ethical issues. Although all of the scientific fields that support these results are merged successfully in archaeology, each one has specific techniques of data collection and processing.

**Fig 2:**
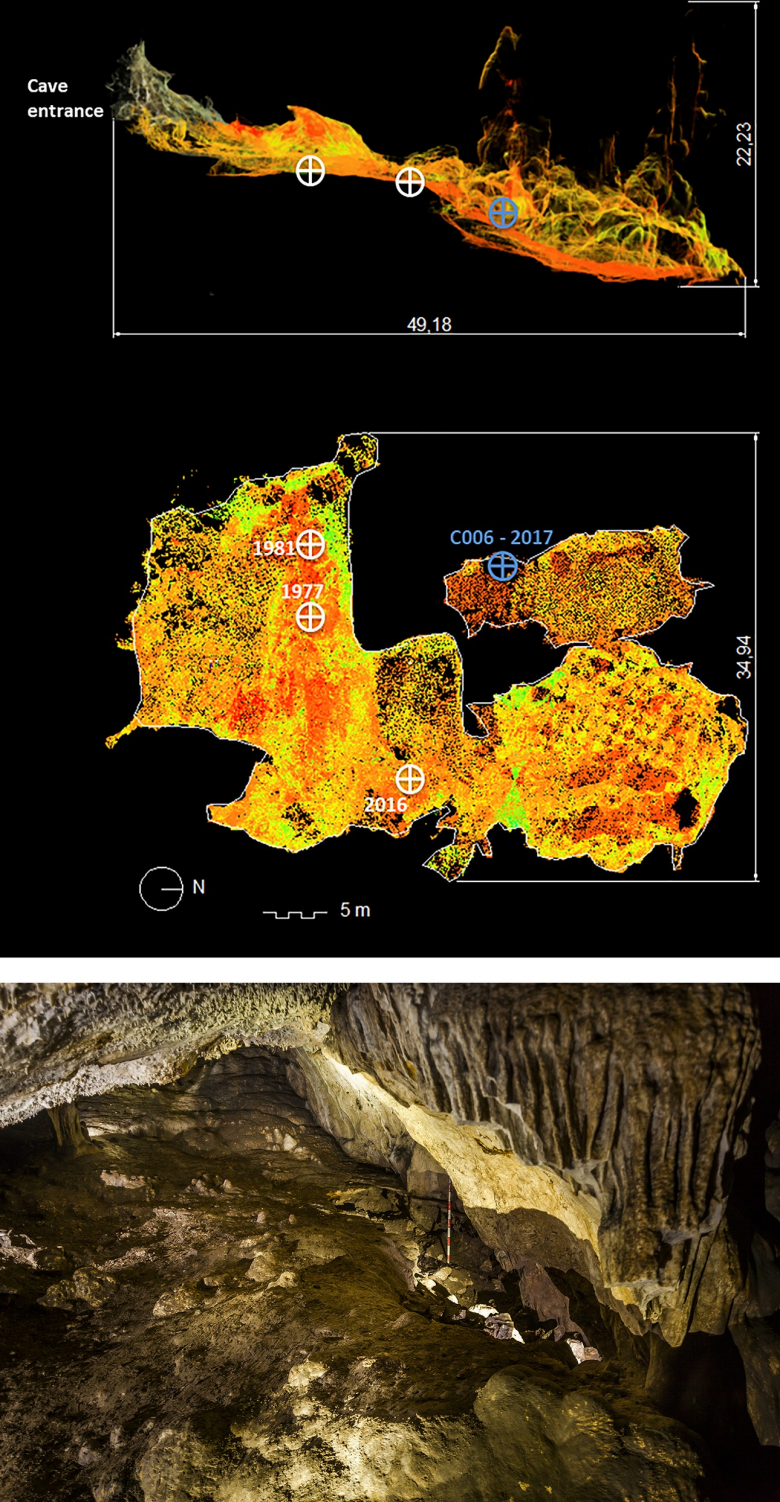
a) Location of Trench C006 on the three-dimensional plan of the cave; and b) Panoramic view from the Centre to the South of Room 4.

The location of trench C006 in the southern part of Room 4 (Fig 2) coincides with an area in which the surface flowrock was broken and absent (Unit 0). The irregular shape of the trench corresponds exactly with the limit of the flowrock. The excavated area is approximately 5 m^2^, with a maximum length of 5 m running parallel to the West wall of the cave and a width of approximately 1 m. Careful excavation confirmed that only the upper layers immediately beneath the surface level were affected by contemporary human and animal activities. Because the stratigraphy of caves usually become a palimpsest subjected to taphonomic processes [22], the excavation proceeded with great care with particular attention to the definition of the contacts between the stratigraphic units [23] and using total stations and laptops with the EDM Mobile software [24] to record topographically every archaeological element. The information was logged in a database easily exportable to storage and data management software, and adapted for the creation of spatial graphic outputs. Both the vertical stratigraphy and the microcontextual relationships of the elements associated with the deposition under study were clearly identified and documented and all of the archaeological materials and samples were duly recorded in their corresponding excavation units. From each and all of the Stratigraphic Units, 20% of the sediment was processed by flotation, and the rest was screened manually (2.5 mm size), with the exception of the samples set aside for specific analyses.

Regarding the human osteological analysis, sex determination based only on the skulls was fraught by the absence of the mandibles. The method applied in first instance was based on the visual assessment of cranial morphological traits [25–28], morphometric variables [29, 30], and the discriminant functions [31] based on an American sample from the Terry Collection. A probabilistic approach, developed by David Navega and based on the European population of the Howells' Craniometric Dataset (n = 317, 153 females and 164 males, estimated sex), was also used. Two types of analysis were conducted using a random forest classification algorithm. Age at death was estimated through the assessment of cranial suture closure and dental wear, following several standard methods [32–36].

Strontium isotope analysis was carried out on both skulls. The basis of the technique is that the strontium isotope ratio of ^87^Sr/^86^Sr varies geographically with geology and that strontium moves into the chemistry of living being from rocks and sediment through the food chain [37–39]. Human tooth enamel is the choice material for analysis [38, 40–43] and the method provides a robust means for examining human mobility in the past. An essential question concerns the local strontium isotope signal for the area in which a burial is found, since levels of strontium isotopes in human tissue may vary from the geological background for a number of reasons [44–46]. It is therefore necessary to measure the bioavailable levels of ^87^Sr/^86^Sr, i.e. those actually available in the food chain, in order to ascertain local strontium isotope ratios. The local bioavailable isotopic signal of the place of burial was determined here from archaeological fauna provided from the cave. In particular, several samples of terrestrial snails (*Otala lactea*) from the same archaeological context *Locus* 2 and from area C004 were used, as well as another sample (*Oryctolagus cuniculus*) from the archaeological area C005 (located to a few meters from of *Locus* 2). A broader dataset of comparative baseline values is also provided by previous work in the region.

The quantification methods applied in the archaeozoological analysis considered the determination of the number of identified specimens (NISP), weight (g) and the minimum number of individuals (MNI). The anatomical identifications (in the case of vertebrate species) and species determination of the osteological and dental elements has followed the specialised reference literature [47–49] and the bone and shell reference collections of the Instituto Andaluz del Patrimonio Histórico and Estación Biológica de Doñana.

The main aims of the pottery analysis, in line with the well-developed framework of archaeological ceramic analyses [50–54], were the assessment and reconstruction of the likely nature of the depositional and taphonomic processes leading to the creation of the assemblage under study, and the typological (formal and decorative) characterisation of the ceramic materials with regards to the fundamental questions of chronological and cultural attribution of the ritual funerary context under study. The quantification of the number of pottery fragments and the individual measurement of their size and weight enabled to fix a series of reference values for the fragmentation of each unit and/or spatial subdivision. Different methods for basic pottery quantification are discussed extensively in the above mentioned sources, and have indeed been an area of foremost interest and debate over many decades [e.g. 55]. After initial descriptive statistics, size was finally retained as the most appropriate proxy variable for the state of fragmentation of the assemblage under study. An exercise in refitting was carried out in addition, with the specific purpose of identifying sherds belonging to the same pots, with or without a direct physical refit. Both approaches clearly highlighted the sharp contrast between the predominant state of fragmentation and vessel representation (mostly single sherds) and that of the scarce yet very significant cases of pots represented by a larger number of fragments and proportion of the vessel.

The stone tools were quantified and analysed in two main groups: knapped and polished stone. The study of the knapped materials followed works on analytical typology for retouch and extraction techniques [56, 57], completing the definition of particular technical attributes [58], as well as the metric description [59]. The knapped material was grouped at the first level as Retouched and Unretouched. The first group includes tools, and retouched blades and flakes; the second group comprises unretouched blades, flakes, atypical fragments, cores, core remains and debitage products, and chunks. Backing, patina and macroscopic use wear was determined by the observation of the edges with a 20x magnifying glass. The typo-technological analysis followed the main syntheses and terminological/lexical proposals for the western [60, 61], southern [62] and eastern [63, 64] regions of the Iberian Peninsula, whilst carrying out the appropriate adaptations to the characteristics of the Dehesilla Cave assemblage.

Traceological analysis was carried out through the combined use of a Leica MZ16A binocular microscope with a range between 10x and 90x and an Olympus BH2 metalographic microscope with a range between 50x and 400x, coupled with a Canon 450D digital camera. Image software (Helicon Focus v. 4.62) was used to obtain completely focused images.

Seeds and fruits were recovered by flotation. Large representative sediment samples were set aside during the excavation process of each stratigraphic unit, specifically one fifth of the total volume of each unit. All of the samples were processed in a flotation machine with a 1 mm mesh collector for the denser materials inside and a 0.25 mm mesh on the overflow for the lighter materials. After flotation both fractions were dried and transferred to the laboratory, where they were processed manually under a 10-15x magnifying glass. The identification of seeds and fruits was carried out in the laboratory of the Institute of History of the CSIC in Madrid, supported by a reference collection and the specialised literature. The denomination of wild taxa followed Castroviejo [65] and that of cultivates followed the binomial classification [66]. Two complementary quantification criteria were used: the ubiquity of each of the taxa and the number of remains.

All anthracological remains larger than 2 mm were considered for analysis. Carbonisation preserves the cellular structure of wood which, observed under the microscope, enables the taxonomic identification of the charred remains [e.g. 67, 68]. This task was performed under a Leica DP2500 incident light microscope. The taxonomic identifications were established through comparison with Schweingruber's tree anatomy atlas [69] and the reference collection of charcoal at the ICArEHB at the Universidade do Algarve.

After presenting all of the data obtained from the depositional context under study (Results), the Discussion offers an overview of the contemporaneous funerary contexts in the Iberian Peninsula dated within the 4800–4550 cal BC date bracket. A comparative analysis is carried out between the different known cases with emphasis on a range of parameters (type of burial, type of structure, burial area and associated structures, placement, orientation, pathologies and grave goods). This enables us not only to contextualise and interpret the new data but also to explore and infer the patterns and traits documented in the ritual funerary practices of this period in the Iberian Peninsula.

## 3. Results

### 3.1. Archaeological context

Trench C006 has enabled the reconstruction of the stratigraphy of Room 4, the inner-most and furthest from the present day mouth of the cave, thus providing a relatively representative image of this area of the cavity. The 2017 excavation documented a complete stratigraphic sequence and enabled a number of observations regarding the characteristics and use of this chamber.

**Fig 3:**
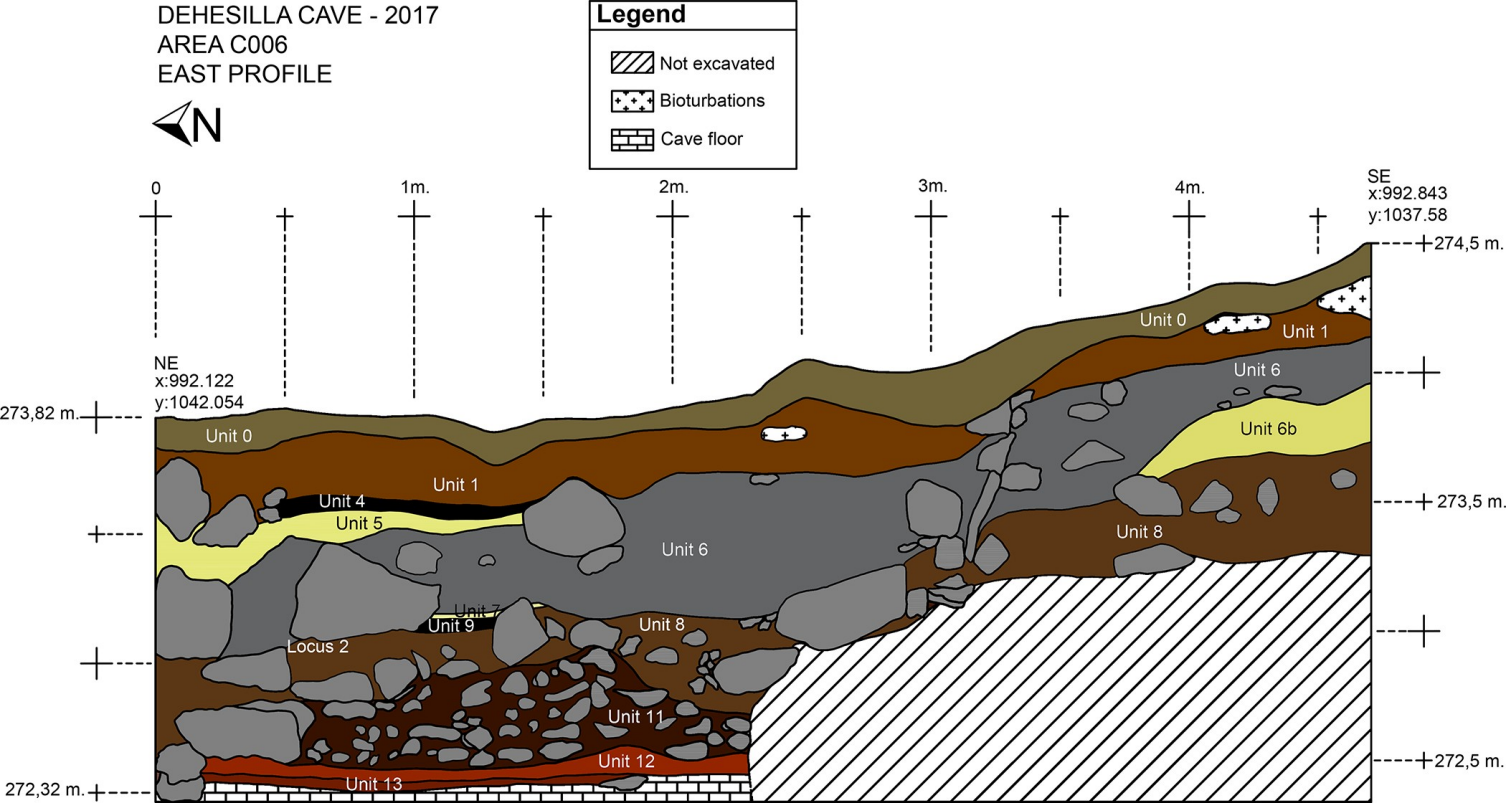
East section of C006.

The sequence includes several thick levels, which appear to correspond to geological events in which rocks, sediments and water may have been carried down from the adjacent Room 2 or possibly through the roof of the cave by means of cracks and chimneys. These levels display a descending south to north slope, from the mouth to the interior of the cave, and form a wedge-shaped accumulation in the southern half of the chamber. The anthropic sequence includes a number of levels, defined on the basis of their stratigraphic characteristics and archaeological materials, belonging to different Neolithic periods (Fig 3). The lower levels date to the Early Neolithic. Of these, the upper level (Unit 8) displays a notable south to north descending slope, and is constituted in great part by medium to large sized limestone blocks, some up to 70 cm. The formation of this level appears to be due to collapse and torrential events.

**Fig 4:**
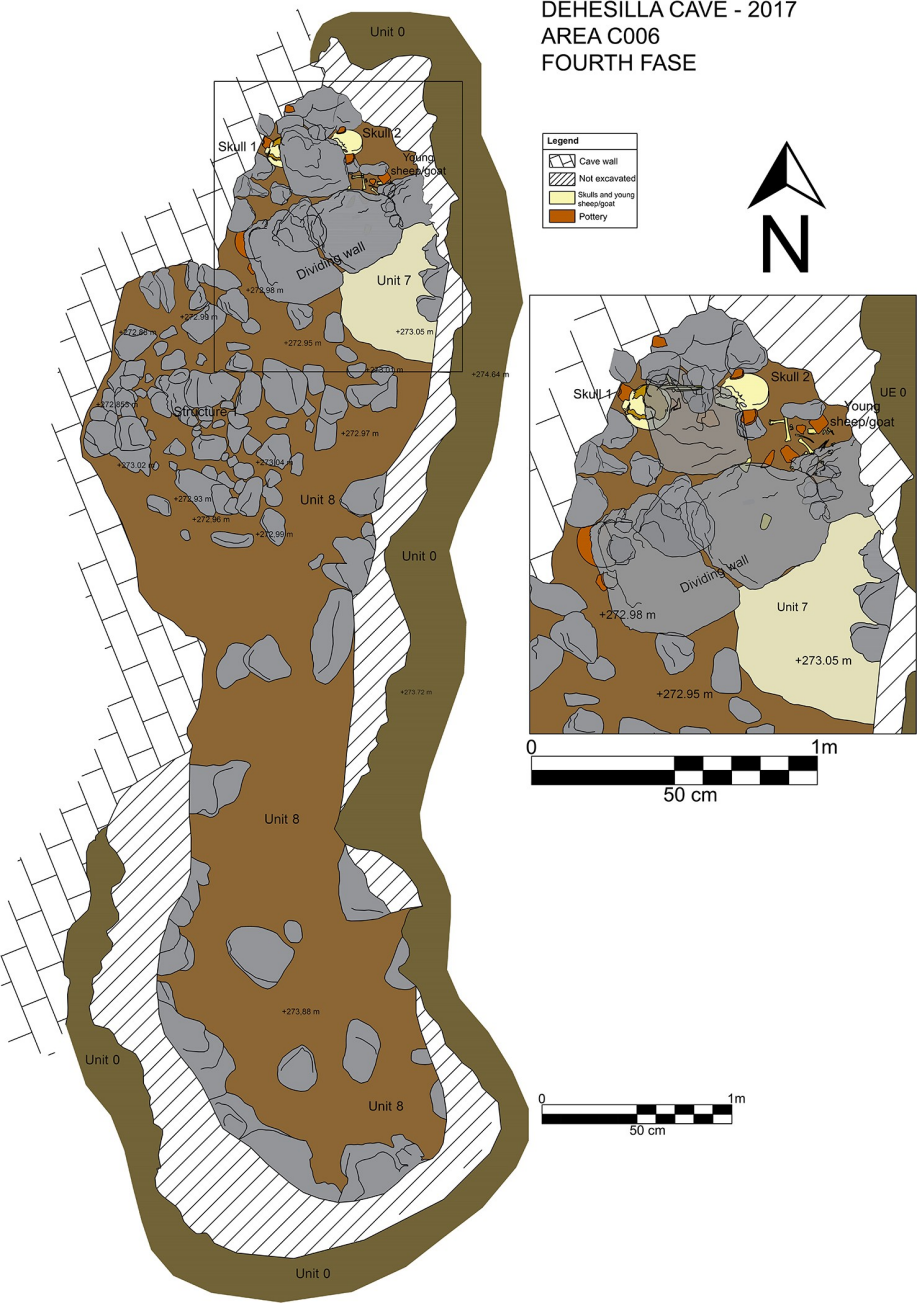
Plan of the fourth stratigraphic phase (Middle Neolithic) in C006.

**Fig 5:**
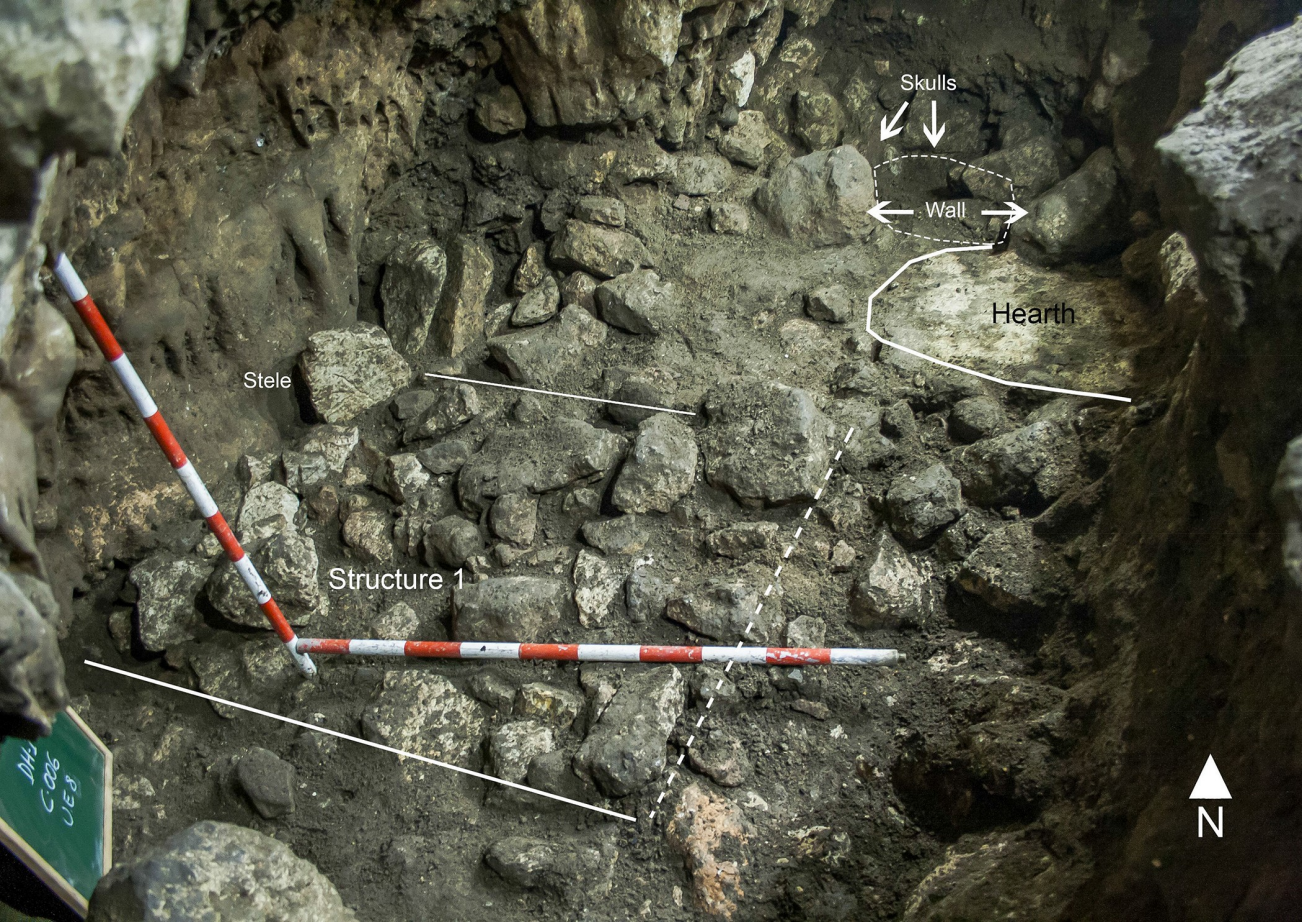
View of the archaeological structures of *Locus* 2 from the North.

The upper contact of Unit 8 is the ground level on which the depositional event analysed in this paper took place, dated at some time between ca. 4800–4550 cal BC and with a material assemblage consistent with the Middle Neolithic (see below). The Units and Structures of interest here are *Locus* 2, the dividing Wall, Platform/Structure 1 and Hearth 7/9 (Figs 4 and 5). *Locus* 2 is the fundamental element of the funerary context (and this name is therefore extended to the association of elements forming the deposition). *Locus* 2 is constituted by two human skulls, without the mandibles, and the main part of a very young sheep/goat, the remains of which were relatively well articulated. The area also yielded a notable pottery and stone tool assemblage. The two skulls were found approximately 20 cm apart, at a very similar level, in a clayey sediment with a medium to low compaction. The closest to the western wall of the cave was partially covered by a limestone block approximately 35 cm in length. The sheep/goat offering was placed by the second cranium. These three elements, carefully placed amongst medium sized limestone blocks, belong to a single depositional event, associated no doubt with the other structures documented in the same area.

To the south of *Locus* 2 an alignment of large stones in an E-W direction was documented, forming a right angle with the western wall of the room. Spatially, these stones create a dividing wall (Wall) between the deposition and two other structures. The first is Platform/Structure 1. To the southwest of the wall, in the central area of the excavated area, where the cave wall forms a kind of natural niche, there is a stone structure, a platform of sorts created by the juxtaposition of stones placed at a very similar level (Platform/Structure 1). The shape of the structure is approximately rectangular, with a maximum length (E-W) of 1,30 m and width (N-S) of 1 m. The outline of the structure, abutted to the West into the wall of the cave, is clearly defined to the North and South but not so clearly to the East. At the northwestern corner, by the cave wall, there was an upright stone placed vertically and outstanding above the other stones of the structure, with a flat face with geological (natural) crossed lines facing the platform.

The second structure to the south of the dividing wall is the Hearth 7/9, constituted by Units 7 and 9. The former is a layer of ash, forming a circular area, approximately 0,40 m N-S and 0,6 m E-W. Its formation must have been relatively rapid. The ash is not compacted (loose), its composition is homogeneous, its colour is beige and its texture is fine. Underneath the ash, Unit 9 is a layer of burnt soil and charcoal. It is dark in colour and granular in texture. It contains a high proportion of charred organic remains. The overlayer of ash spreads slightly beyond the underlying burnt layer towards the South. To the North the hearth is delimited by the above-mentioned dividing wall.

These structures, documented in close proximity to one another in the northern half of trench C006, constitute the spatial elements of a depositional context in relation to a funerary practice of ritual nature, with no indication of continued customary usage of this room of the cave.

### 3.2. The human remains

The human remains of *Locus* 2 comprise two crania (skulls without mandibles), identified as Cranium 1 (find number C006-118) and Cranium 2 (find number C006-124), a single tooth (a right permanent mandibular canine) and a thumb distal phalanx.

**Fig 6:**
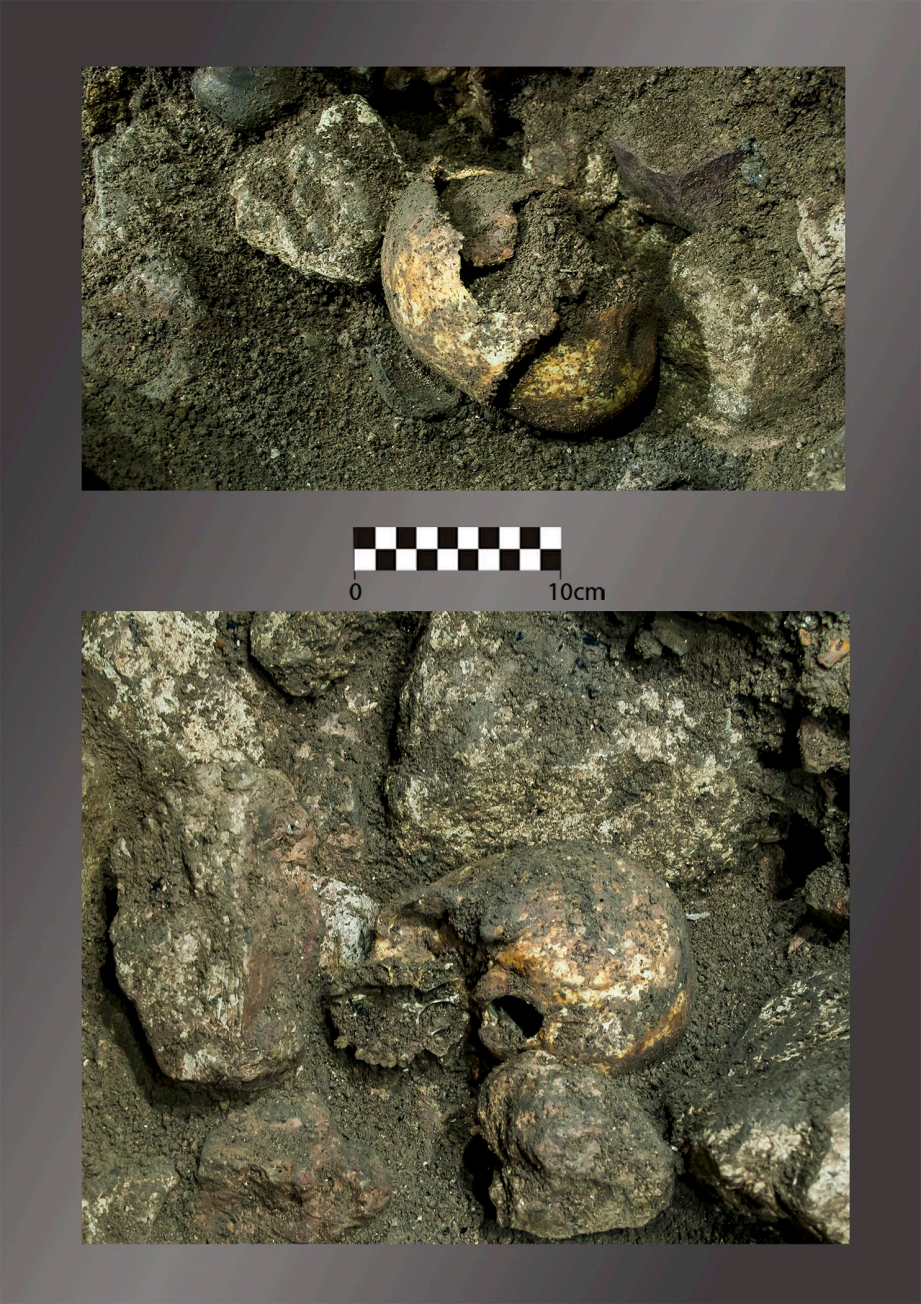
*In situ* image of Cranium 1 (top) and Cranium 2 (bottom).

The first cranium to be identified (Cranium 1) was found partially under a large white rock, approximately 10 cm from the wall of the cave (Fig 6). It was lying on its right side facing North. The left parietal bone collapsed through the sagittal and lambdoid sutures. The anterior and lateral region of this bone was absent, damaged post-mortem. The frontal bone and other bones of the face also gave in through the coronal suture, probably due to the pressure of the stone above. The face was lying partly over another rock. While removing the sediment in order to uncover the cranium and to look for possible anatomical connections that did not exist, a second cranium was identified approximately 20 cm to the East of Cranium 1, also surrounded by rocks. This cranium (Cranium 2) was complete and was lying on its right side, facing West in the direction of the first skull.

**Fig 7:**
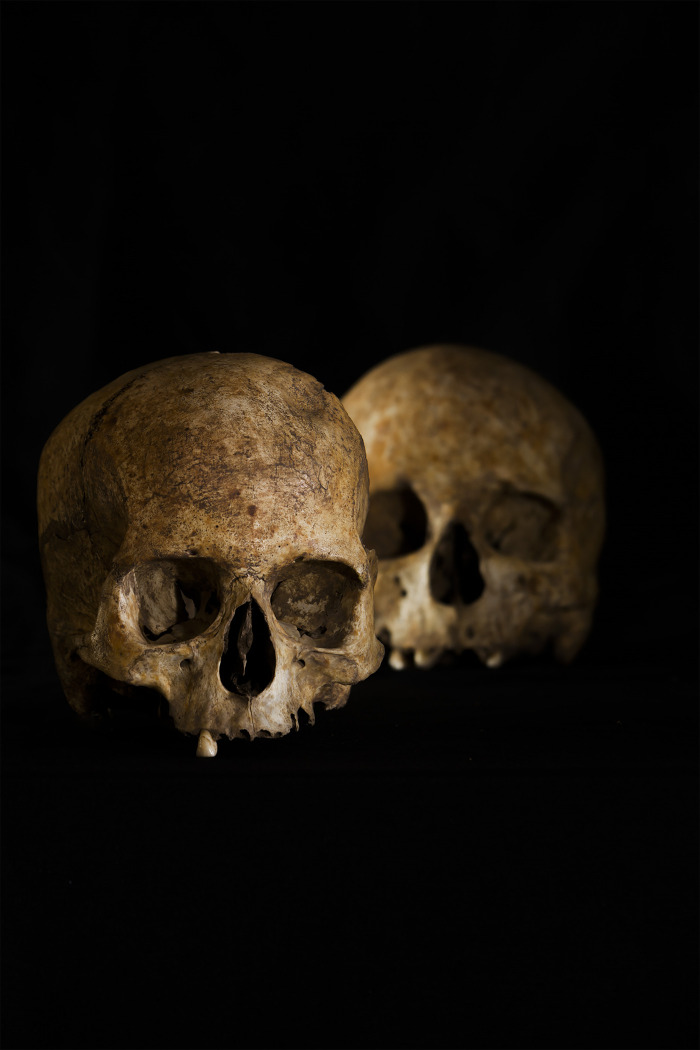
Laboratory image of the skulls (1: front; 2: back).

Sex determination of these two individuals is very problematic (Fig 7). The absence of the pelvic bone, the most reliable indicator for sex determination, and the lack of reference data for the sexual dimorphism of the population to which they belonged, associated with the absence of typically female or male cranial traits prevents a definitive diagnosis.

As several authors have pointed out [25,70–72] sexual dimorphism varies between populations, and it is impossible to establish definite morphological and metric boundaries between males and females [25]. Following the methods based on the visual assessment of morphological cranial traits [25, 26, 28], Cranium 1 may have belonged to a female individual and Cranium 2 to a male. However, the form of the supraorbital margin of both crania, according to Graw et al. [27], suggests two males, although the use of a single trait is not reliable, as highlighted by Bruzek and Murail [71].

Metric analyses are also problematic since they are even more population-sensitive than the morphological traits described above, and it is generally accepted that metric standards should not be used in populations other than the ones of which they were developed [25]. Aware of this problem, sex assessment through morphometric traits was nonetheless considered as an alternative method. Attending to the mastoid length [29] both crania classify as males, with 28.33 mm for Cranium 1 and 28.76 mm for Cranium 2. Based on the foramen magnum [30], Cranium 1 is classified as female, while Cranium 2 may be classified as male, based on the maximum length and circumference, or as female based on the maximum width and circumference. To avoid the use of a single trait, the discriminant functions [31] were applied and, interestingly, the results coincide with those of the morphological analysis, classifying Cranium 1 as female and Cranium 2 as male. In addition, a probabilistic approach based on the European populations of the Howells' Craniometric Dataset was also applied. Two types of analysis were conducted based on a Random Forest classification algorithm as the underlying sex estimation model: 1) Using the raw craniometric variables, focusing on cranial size; 2) Computing new variables through the scaling of the craniometric variables by the geometric mean of the measurements available for each individual. The sex estimation model was built on the scaled craniometric measurements and the geometric mean of the raw measurements. This procedure provided an approximate representation of the cranial form, including parameters of both size and shape. As a result, Cranium 1 offers a probability of 0.65 of being female, attending to size only, and a probability of 0.74 of being female when size and shape are taken into account. Cranium 2 has a probability of 0.520 of being male considering size only. The probability of being male increases to 0.742 when size and shape are put together, suggesting a male individual with a small cranium. These probabilities offer no definite solution, but they are in line with the results obtained from the morphological analysis. However, sex diagnosis should only be considered reliable in cases of a probability higher than 0.95 [71].

Considering the results, Cranium 1 probably belonged to a female individual and Cranium 2 to a male. This suggestion should however be considered as preliminary, until there may be a larger available sample that may allow a better understanding of the sexual dimorphism of the population to which these two individuals belonged or until genetic analysis may be performed.

Age at death was estimated through the assessment of cranial suture closure and dental wear. The obliteration of the spheno-occipital synchondrosis confirms the presence of two adults. Cranium 1 presented all sutures opened, which in accordance to Meindl and Lovejoy's method [35] indicates a young to middle-aged individual, with an age at death between 18 and 45. Following Masset [36], the interval obtained is slightly wider, between 24 and 55 years of age. As this cranium does not preserve the molar teeth, the only method that could be applied based on dental attrition was that proposed by Lovejoy [34] which points to an individual aged between 24 and 35. Cranium 2 belonged to an older individual. Based on the obliteration of the lateral-anterior sutures [35] the age at death of this individual was between 24 and 49, or between 24 and 60 based on the vault sutures. Masset's method [36] provides an age interval between 41 and 70. The assessment of dental wear, in this case, suggests lower age ranges: 30–40 [32], 25–35 [33] and 35–40 [34].

Neither cranial suture closure nor dental wear are considered the best methods to estimate age at death, even though dental wear is accepted as a more reliable method. Based on the above, and considering all of the age ranges obtained, age at death may be suggested for Cranium 1 between 24 and 40 years, and for Cranium 2 between 30 and 50.

**Fig 8:**
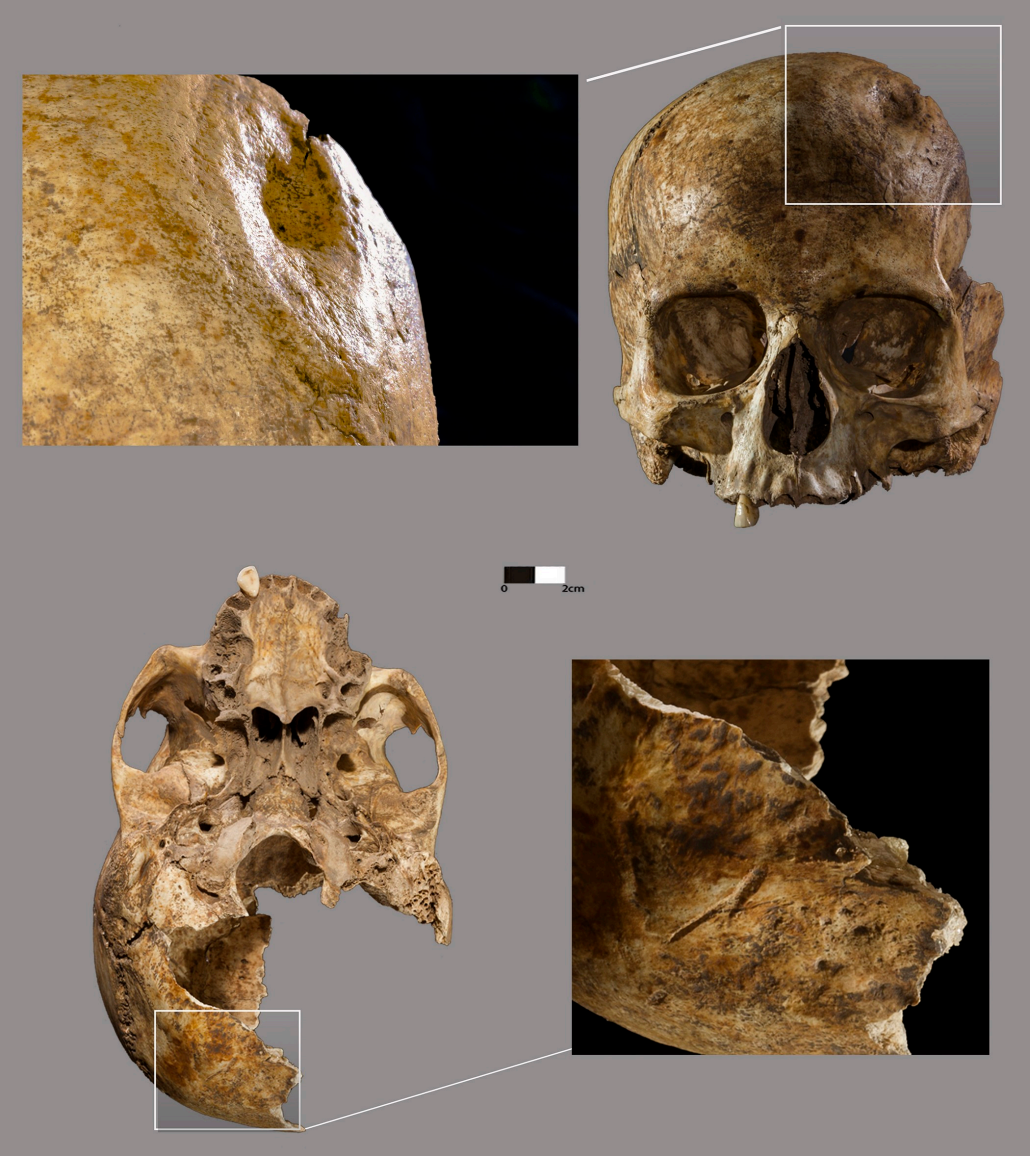
Lesion and cut marks on Cranium 1.

The most striking pathological evidence was observed on Cranium 1: a depression on the left side of the frontal bone at about 30 mm of bregma and near the coronal suture, with clear signs of osseous healing, with bone remodeling (Fig 8). The depression is oval in shape with a maximum anteroposterior diameter of about 56 mm and a mediolateral diameter of 35 mm. From the outer to the inner part of the lesion, on the medial margin, a flattened and smooth area about 5 mm in width is observed which extends along a line on the anterior and lateral margins, followed by a pronounced, more inclined smooth area of about 5 mm width on the medial side that loses its definition on the anterior side, becoming more irregular but of greater width (12 mm), while on the lateral side there is a decrease of its slope, keeping the width observed on the medial side. The more depressed area is about 19 x 12 mm. There are no signs of bone reaction in the inner table, just a slight depression that apparently corresponds to the medial external margin of the lesion.

Blunt trauma was initially considered as the cause, however the absence of radiating fracture lines and the observation on the contours of the lesion of three distinct areas of smoothing may be indicative of an incomplete trepanation by scraping. This cranium also presents two cut marks on the occipital region, with a length of about 18 mm, with its anterior extremity at a distance of approximately 55 mm from the lambda and 52 mm from the asterion.

Both crania present signs of non-active porotic hyperostosis, a physiological stress indicator, more evident on Cranium 2 on the frontal, parietal and occipital bones. On Cranium 1, these lesions are more intense on the anterior region of the parietals. Regarding its etiology, while some authors [73–75] consider iron deficiency anaemia as the most probable cause, others [76, 77] point to haemolytic and megaloblastic anaemias. Cranium 1 also displays three button osteomas, an asymptomatic benign and slow-growing osteogenic tumour [78] on the right parietal bone. One, with mediolateral and anteroposterior diameters of 9 and 5 mm, respectively, located at 24 mm from the coronal suture; another in a more posterior and lateral position about 10 mm from the first, round in shape with a 5 mm diameter, and the last one on the parietal posterior region, at about 23 mm from the sagittal suture and 21 mm from the lambdoid suture, with approximately 8 mm in mediolateral diameter and 7 mm in anteroposterior diameter.

#### 3.3. Isotope analysis

**Fig 9:**
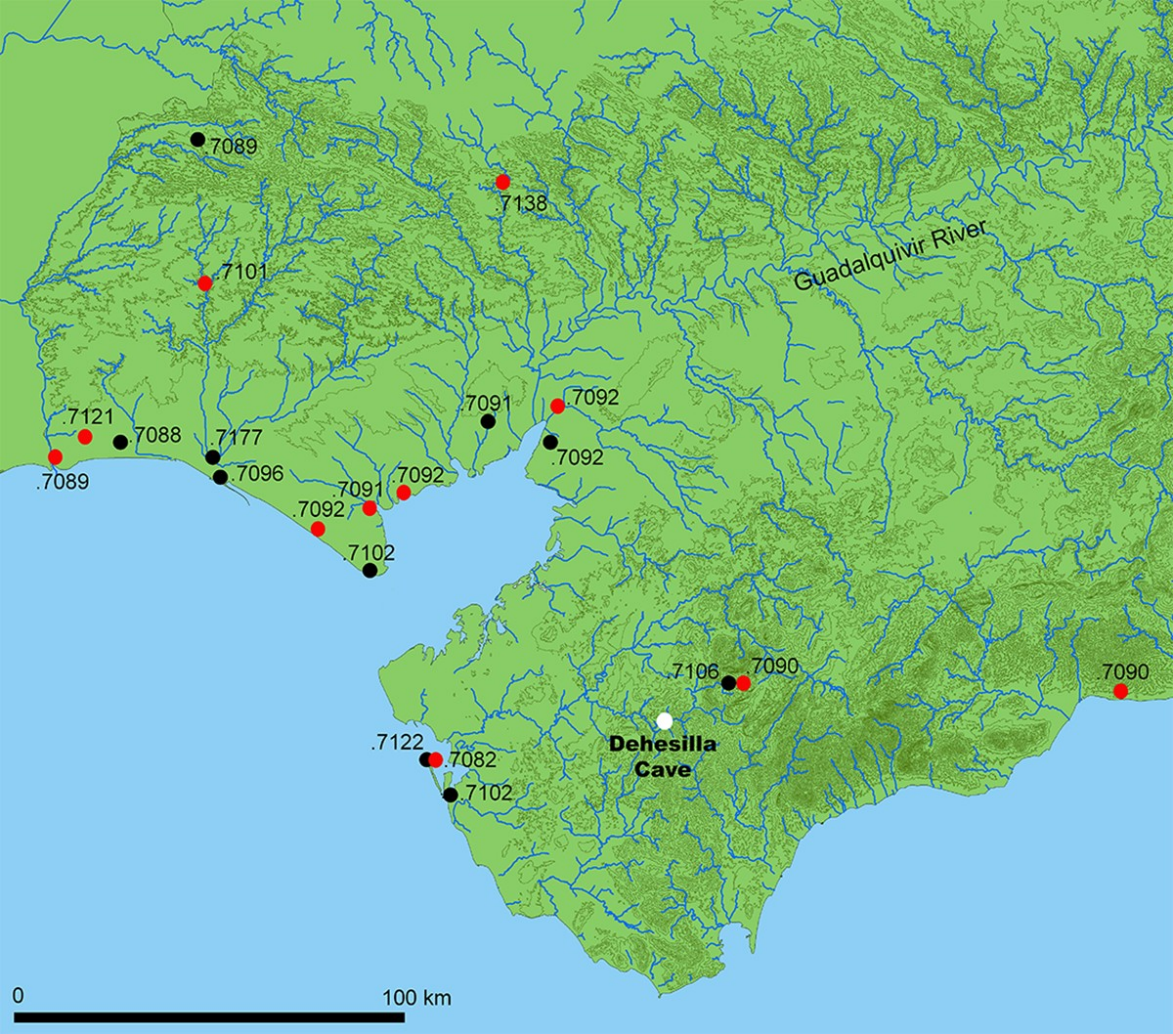
Map of Western Andalusia with the location of strontium isotope reference samples. Black dots are human remains; red dots are plants and animals (Theme map bases: [21]).

Strontium isotope values have previously been measured in a number of samples from Andalusia, including both human and faunal remains. A map of the location of the samples and their average ^87^Sr/^86^Sr values provides some indication of the regional variations (Fig 9) in accord with the three main geological units of Southern Iberia: the Iberian Massif, the southern Baetic Cordillera and the Guadalquivir Basin. Floral and faunal samples are distinguished from human enamel samples, which are not considered as reliable baseline values due to possible residential mobility.

**Fig 10:**
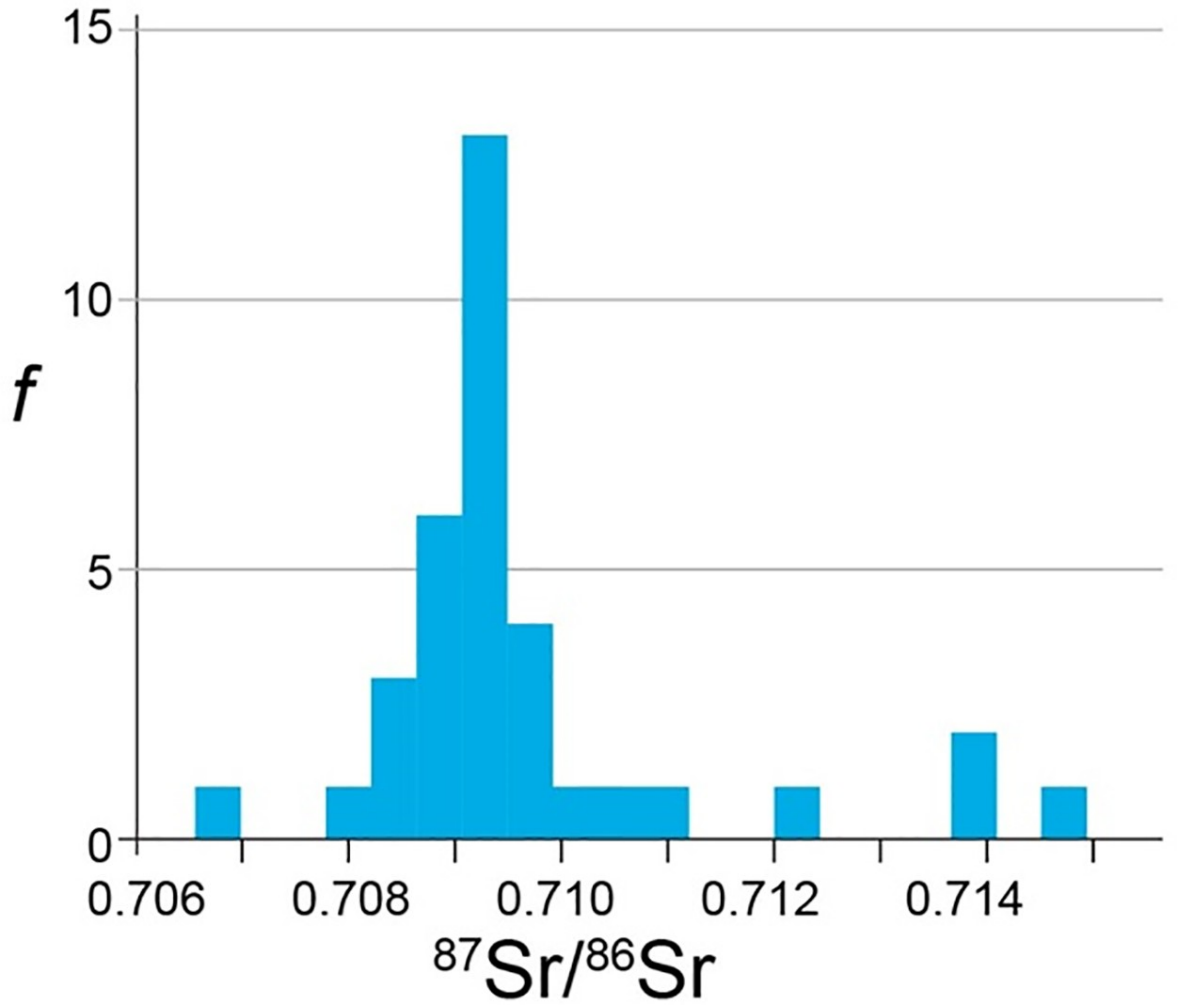
Histogram of strontium isotope ratios from plant and animal remains from Andalusia.

A histogram of all 43 values for bioavailable plant and animal remains is presented in Fig 10. The range of values extends from a minimum 0.7067 to a maximum 0.7147. The mean of this distribution is 0.7095 ± 0.0014. Thus, the bioavailable values for much of Andalusia fall between 0.7081–0.7109, with some higher values recorded locally.

The geology of the immediate area around Dehesilla Cave is complex, composed of both ancient and more recent geological deposits. The cave itself formed in Jurassic limestone and dolomite. Around the entrance to the cave there are deposits of Keuper marls, but the larger area around the site is dominated by Cretaceous limestones and derivative sands and clays. According to estimates based on ^87^Sr/^86^Sr in ancient sea water, the baseline values for these geological formations should be in the order of 0.7070–0.7080 [79].

**Fig 11:**
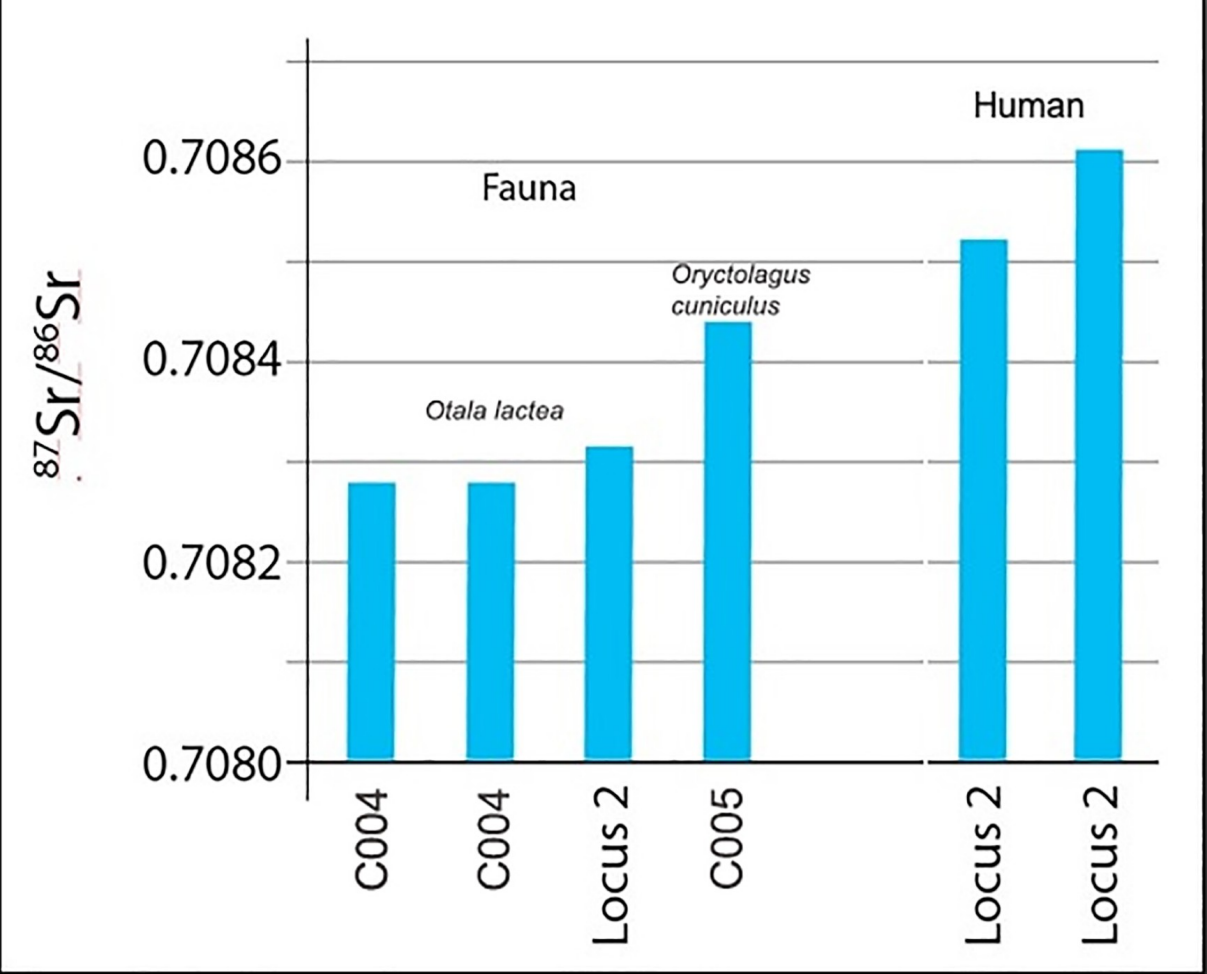
Bar chart of fauna and human values from Dehesilla Cave.

Bioavailable strontium isotope ratios were measured directly on faunal remains recovered from the archaeological deposits of the cave. Four samples of rabbit bone and terrestrial snail shell (the latter from *Locus* 2 itself) were analysed in order to provide a precise local baseline. These values are shown in Table 1 and Fig 11 and cluster tightly between 0.7083 and 0.7085.

**Table 1:** Results of Sr isotope analysis.

Lab Code	Dehesilla ID	Context	Species	Sample	^87^Sr/^86^Sr
F10290	DH17-22	C006-*Locus* 2	*Homo*	Skull 1 –PM1 Right	0.708371
F10291	DH17-23	C006-*Locus* 2	*Homo*	Skull 2 –PM2 Right	0.708509
10295	DH17-29	C006-*Locus* 2	*Otala lactea*	Terrestrial snails	0.708304
F10292	DH17-26	C004-Unit 9	*Otala lactea*	Terrestrial snails	0.708298
F10293	DH17-27	C004-Unit 14	*Otala lactea*	Terrestrial snails	0.708264
F10294	DH17-28	C005-Unit 3	*Oryctolagus cuniculus*	Tibia	0.708431

The values obtained from the *Locus* 2 human samples are indicated on the same plot. The range of values exhibited by the samples from the two skulls, between 0.7083 and 0.7086, is very narrow, coinciding neatly with the baseline values provided by the faunal remains from the site. The range of values from the human and faunal samples is also a good match to the expected strontium ratios of the limestone landscape of the broader area of the sedimentary and Neogene Basins. These observations make it very likely that the human individuals represented by the remains were local to southwestern Andalusia, and quite probably to the site itself or immediate surrounding area.

### 3.4. Grave goods

#### 3.4.1. Animals

**Fig 12:**
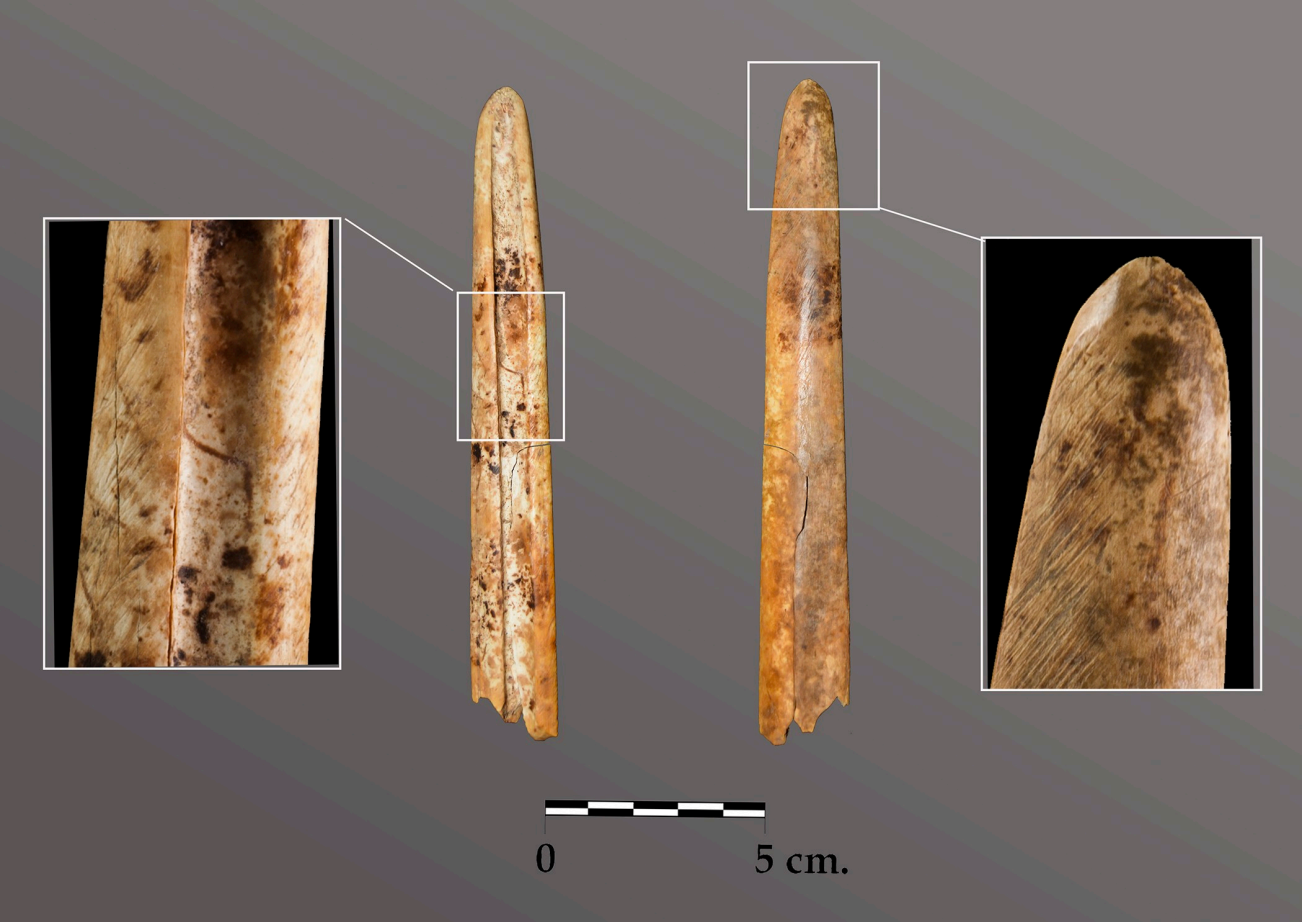
Bone spatula made from a deer metatarsal, with extensive use wear.

A relatively large animal bone assemblage was recovered from *Locus* 2 (Table 2), as well as the remains of naturally ocurring microvertebrates and moluscs. The animal remains have been taxonomically determined as belonging to *Cervus elaphus*, *Sus* sp., *Ovis aries/Capra* sp., *Oryctolagus cuniculus*, *Otala lactea*, *Unionidae*, as well as indeterminate remains of fish and shell, a turtle and other vertebrate and invertebrate species. This assemblage displays a fragmentation similar to that of the other stratigraphic levels in C006, in such as way that these remains may not be presumed to have been deliberately included in the depositional event. The presence of two freshwater molusc shells (*Margaritifera auricularia*) and a perforated marine molusc shell (*Acanthocardia aculeata*) upon Structure 1 are noteworthy exceptions, as is a bone tool (find number C006-120) placed between the two skulls. This well-worked spatula with notable abrasion marks from use was created on a deer metatarsal (Fig 12).

**Table 2:** Number of Identified Specimens (NISP) and Minimal Number of Individuals (MNI). The *Ovis aries*/*Capra* sp. Line includes the remains of the infantile sheep/goat found in *Locus* 2. There is no secure anatomical characteristic to distinguish bones from *Ovis aries* and *Capra* sp. The Class II mammals are those with a body mass between 18 and 200 kg [80, 81] according to the decay processes of mammals in Mediterranean ecosystems.

Taxa		*Locus* 2	Structure 1	Unit 7	Unit 9
*Ovis aries /Capra* sp.	NISP	49		1	
	MNI	2		1	
*Cervus elaphus*	NISP	1	1	1	
	MNI	1	1	1	
*Sus* sp.	NISP	3		1	
	MNI	1		1	
*Oryctolagus cuniculus*	NISP	8		9	1
	MNI	2		2	1
Freshwater turtle	NISP	4			
	MNI	1			
Fish	NISP			1	
	MNI			1	
Class II mammals	NISP	5		11	
Indeterminate	NISP	30		46	1
TOTAL	NISP	100	1	70	1

**Fig 13:**
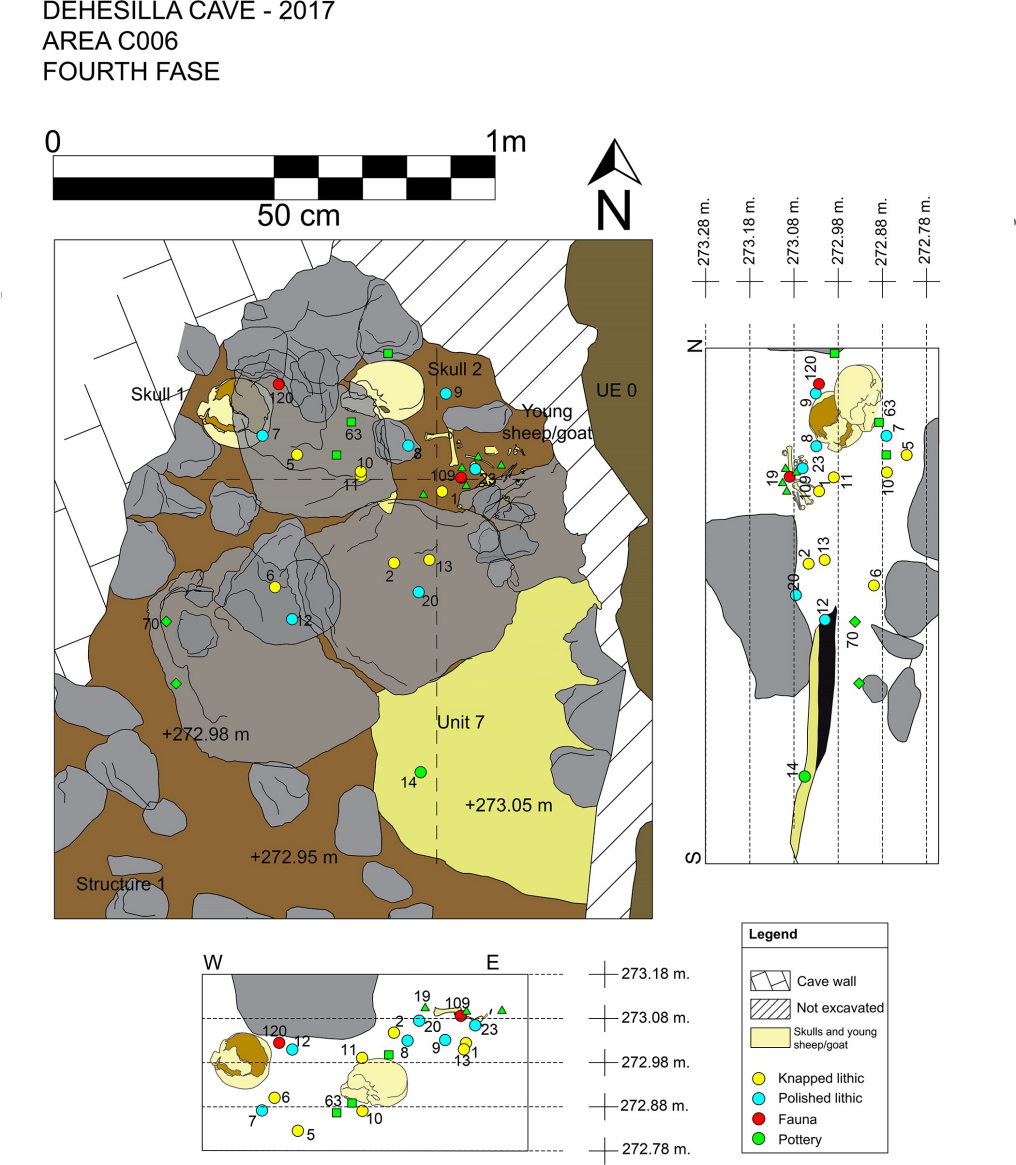
Plan and sections of *Locus* 2 with a precise spatial location of the offerings. The two sections correspond to the perpendicular dashed lines indicated on the plan.

**Fig 14:**
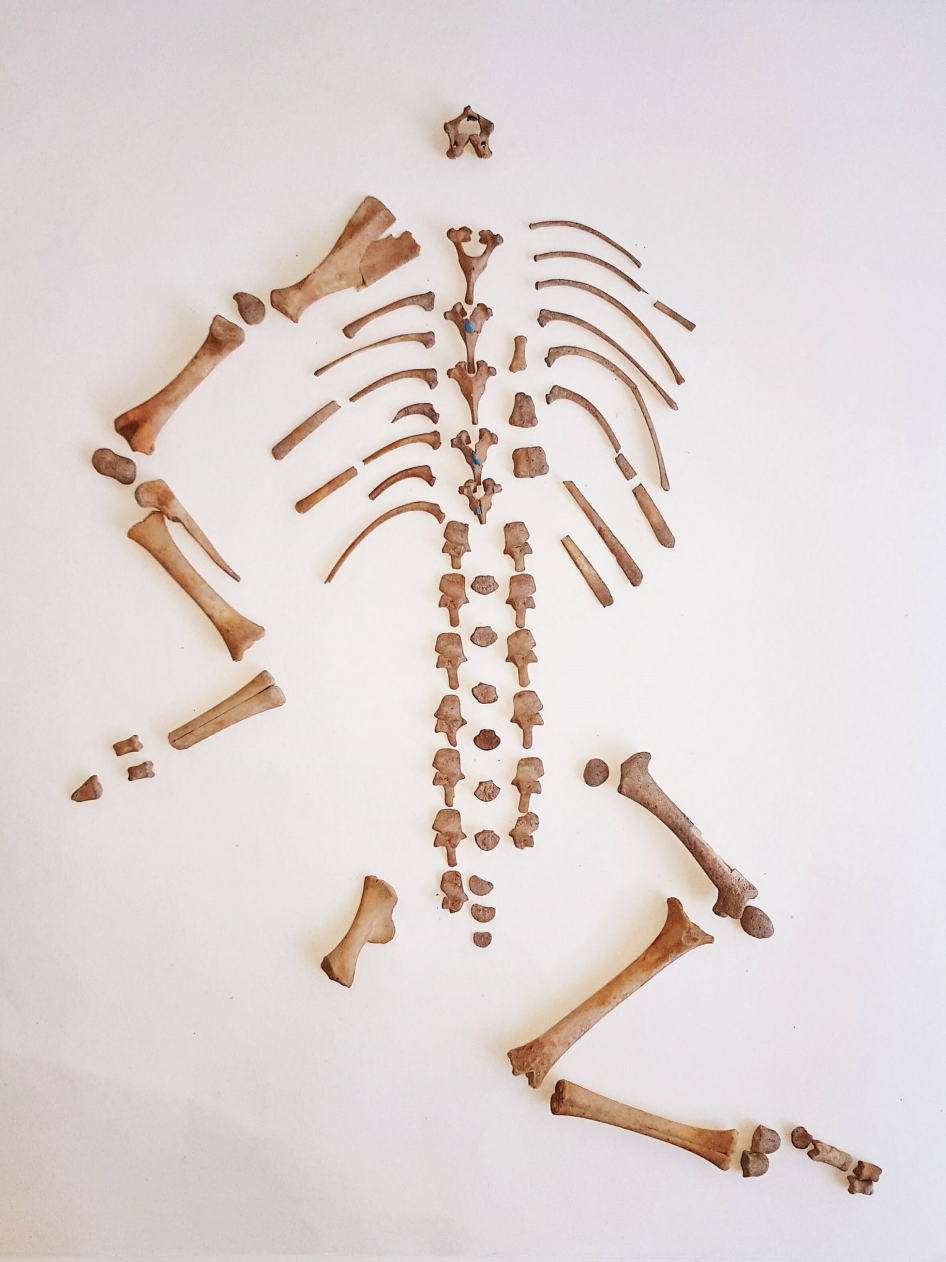
Laboratory image of the young sheep/goat skeleton.

To the east of the skulls (Fig 13, find number C006-109) the remains of an infantile sheep/goat individual were found, which must have been sacrificed and deposited complete or near complete, as indicated by the elements in anatomical connection and order. No cut marks, tooth marks or thermoalterations were identified (Fig 14). Interestingly, not all of the skeletal elements were recovered. Specifically, the anterior right and posterior left extremities and the skull were absent (Table 3). The absence of six cervical vertebrae, seven thoracic vertebrae and the sacrum are also noteworthy.

**Table 3:** Anatomical remains of the *Ovis/Capra* skeleton found in *Locus* 2.

Bones	Left	Right
Cervical vertebra	1	
Dorsal vertebrae	5	
Lumbar vertebrae	7	
Ribs	7	6
Rib fragments	2	
Sternum	3	
Scapula	1	
Humerus	1	
Radius	1	
Ulna	1	
Metacarpus	1	
Pelvis	1	
Femur		1
Patella		1
Tibia		1
Metatarsus		1
Phalanx I	1	
Phalanx II	4	
Phalanx III	1	
TOTAL	47	

The taxonomic identification of the skeleton is complex. The presence of both *Ovis aries* and *Capra hircus* is known in contemporaneous and earlier levels, and therefore the sacrificed animal could belong to either of these two species or to *Capra pyrenaica*. The state of the fusion of the thoracic and lumbar vertebrae may indicate an age at death of less than ten days, following the parameters set out in a study of the Aragonese sheep breed [82]. According to this author, the arch of the thoracic vertebrae fuses before birth and the arch of the lumbar vertebrae within 10 days after birth.

The age estimate of the animal may be of interest in relation to the approximate time of year at which the ritual deposition took place, although naturally this is subject to the uncertainties regarding the possible changes in the reproductive cycles of these species over time. At present, wild Caprinae usually have spring-time lambing seasons, when pastures are most abundant [83]: *Capra pyrenaica* (May-June), *Capra aegagrus* (April-May) and *Ovis gmelini* (May). In contrast, many present-day Iberian domestic sheep and goat breeds can give birth at any time of the year [84–86]. Artificial selection and productive interests have led to a near-continuous ovarian cycle [87] which, in some close modern breeds, for instance the Churra Lebrijana sheep, enables deliveries throughout the year. In other breeds the reproductive cycle has been intensified to reach more than one annual birth, or in others, for instance the Canarian sheep, births are concentrated before Christmas in order to suit consumer demand. However, in sheep breeds such as the Chamarita, in extensive exploitation systems, births are concentrated between May and June; in the Castellana breed between February and March; in the Charmoise breed, 75% of births occur between March and April; and in the Alcalarreña breed they are concentrated between February and March [84]. With the modern management of goat herds, it is also possible to reproduce these animals throughout the year, although for some the highest frequencies of births are concentrated in particular seasons [86]. For instance, for the Payoya goat, an autochthonous Andalusian breed from the same area as the archaeological site, 85% of births are concentrated between August and November and between December and February, for the Blanca Serrana Andaluza breed between March and April or for the Negra Serrana in Spring or Autumn [85]. If we assume that the Neolithic domestic sheep and goat had reproductive cycles marked by the environment of the region, the animal could have been sacrificed and offered ritually coinciding perhaps with the beginning of Spring. This seems to be the case for other Iberian Neolithic archaeological sites [88], although this particular point must remain open, since greater knowledge is needed on the human selective pressures on Iberian Neolithic herds.

#### 3.4.2. Pottery

The pottery assemblage is fairly large, especially in the immediate environment of the skulls (*Locus* 2) and the hearth (Units 7 and 9). However, an important issue concerns the inclusion of pottery vessels in the deposition, and their use in specific activities. Answering this question requires a detailed analysis of the general patterns of fragmentation and representation in the depositional units of interest here (Table 4). Based on the quantitative evidence, two processes of entry into the archaeological record can be proposed for the pottery record. Indeed, most of the assemblage displays a very high fragmentation, and a very low representation, that is, most pottery records correspond to single fragments from different vessels. In Unit 7, impressed, incised and engraved decorative techniques coexist without any clear pattern. It should be noted that these pottery fragments are not usually burned or altered by fire. Meanwhile, in *Locus* 2 there is a greater frequency of engraved decoration. The relative importance (frequency) of engraved pottery in the units conforming the ritual deposition must be noted, as well as the coexistence of two well-known products for the site, impressed and incised pottery, with different technological and stylistic characteristics. In any case, it seems that a large part of the fragmentary pottery assemblage may not be directly associated with the ritual funerary event.

**Table 4:** Summary of the pottery assemblage fragmentation patterns (individual fragment size range and average). Excluding the partially reconstructed vessels (see Table 5).

Unit	Nº of fragments	Size Range	Size Average
UE 7	76	1,5–8 cm	3,6 cm
UE 9	8	2–8,5 cm	4,3 cm
UE 8 *Locus* 2	171	1–8 cm	3,5 cm

**Fig 15:**
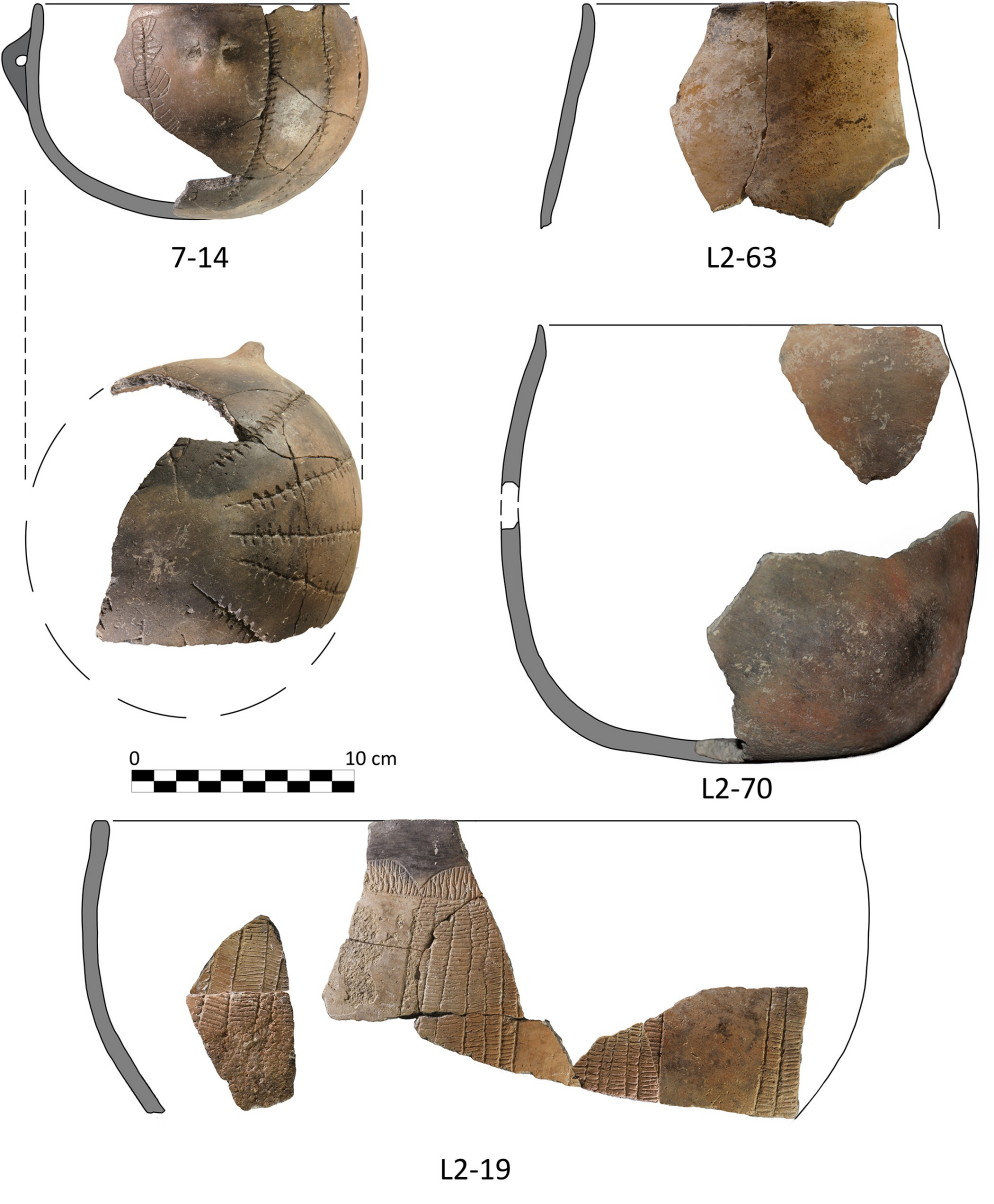
Pottery vessel offerings or burial goods from *Locus* 2.

**Fig 16:**
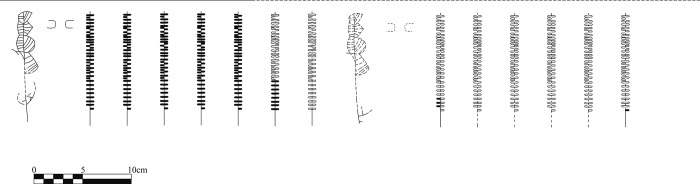
Schematic ideal reconstruction of the decoration of vessel 7–14.

In contrast, there is a smaller set of fragments belonging to four separate vessels, for which a relatively larger proportion is preserved, and that appear to have been deliberately deposited at the time of the ritual (Table 5). First of all, there is a group of 10 fragments linked to Unit 7, the upper layer of the hearth, with physical connections between all the fragments (Fig 15, Id. 14). The conjoining fragments enable the reconstruction of approximately one-third of a closed, fine-walled bowl with a high quality surface treatment and unique decoration within the Dehesilla pottery repertoire known to date. Specifically, it is decorated with an engraved and staged schematic motif. It has 11 or 12 radial lines arranged from the base, with a possible progressive evolution of the initial design. At least one of the radial lines structures a series of semi-circular shapes, smaller in the upper area (below the rim edge) and larger in the central area of the bowl, filled with horizontal or oblique parallel lines (Fig 16). The physical relationship of all the recovered fragments with no 'orphan' sherds suggests breakage with little subsequent dispersal of the fragments.

**Table 5:** Fragmentation and representation of the intentionally deposited pots.

Pottery Record Id.	Nº of fragments	Maximum preserved vertical measurement	Total Weight
Hearth Unit 7 –Id. 14	10	9.5 cm*	202 g
*Locus* 2 –Id. 19	8	13 cm	165 g
*Locus* 2 –Id. 63	5	10 cm	194 g
*Locus* 2 –Id. 70	2	19.5 cm*	408 g

* Complete vessel height.

Secondly, there is a set of 8 fragments found in *Locus* 2 that were directly associated with the sheep/goat described above, and belong to part of a large bowl with a burnished inner surface (Fig 15, Id. 19). It preserves a maximum reconstructed vertical measurement of 13 cm. The preserved portion of the bowl displays a schematic engraved decoration: a band of repetitive semi-circular shapes under the rim upon a horizontal line from which vertical areas alternating smooth spaces and spaces formed by several vertical bands (up to 6 observed in the preserved fragments) filled with parallel horizontal lines are delimited. In contrast, the vertical fill of the semi-circles appears to be deliberately criss-crossed, drawn towards the rim. In two conjoining fragments the outer line of the vertical band is highlighted by small excised triangles. The vessel shows an interesting possible firing effect, with a blackish edge to contrast with a light brown body. Many of the conjoining fragments were found in proximity to each other and to the infantile animal skeleton (Fig 13, Id. 19 and 109, respectively). Therefore, a direct relationship can be inferred between the deposition of this vessel and that of the possible sacrificial sheep/goat.

Thirdly, a set of 5 fragments has also been documented in *Locus* 2, 4 of them rim sherds, representing over 35% of the circumference of the mouth, but with a maximum preserved vertical measurement of only 10 cm, equivalent to the rim and neck of an apparently undecorated pot (Fig 15, Id. 63). Three other fragmentary and amorphous sherds share very similar physical characteristics, but lack a physical connection. They appear all to belong to a plain, truncated inverted cone necked vessel, with even compact walls (6 mm) and very smooth burnished surfaces. Some of the fragments are also partially coloured in black, probably altered by fire. Fig 13 shows the position of the fragments in the immediate vicinity of the male skull (Cranium 2).

Finally, two fragments of the rim and body and base of a single container were documented in *Locus* 2 and were associated with the dividing wall between the skulls and the hearth (Fig 15, Id. 70). This pot is bucket-shaped, with subvertical walls, a flat base, and no decoration. It is tentatively included in the group of outstanding pottery vessels because of the size of the fragments, which is much larger than the average for these units, and the preservation of the near-complete section.

#### 3.4.3. Stone tools

**Fig 17:**
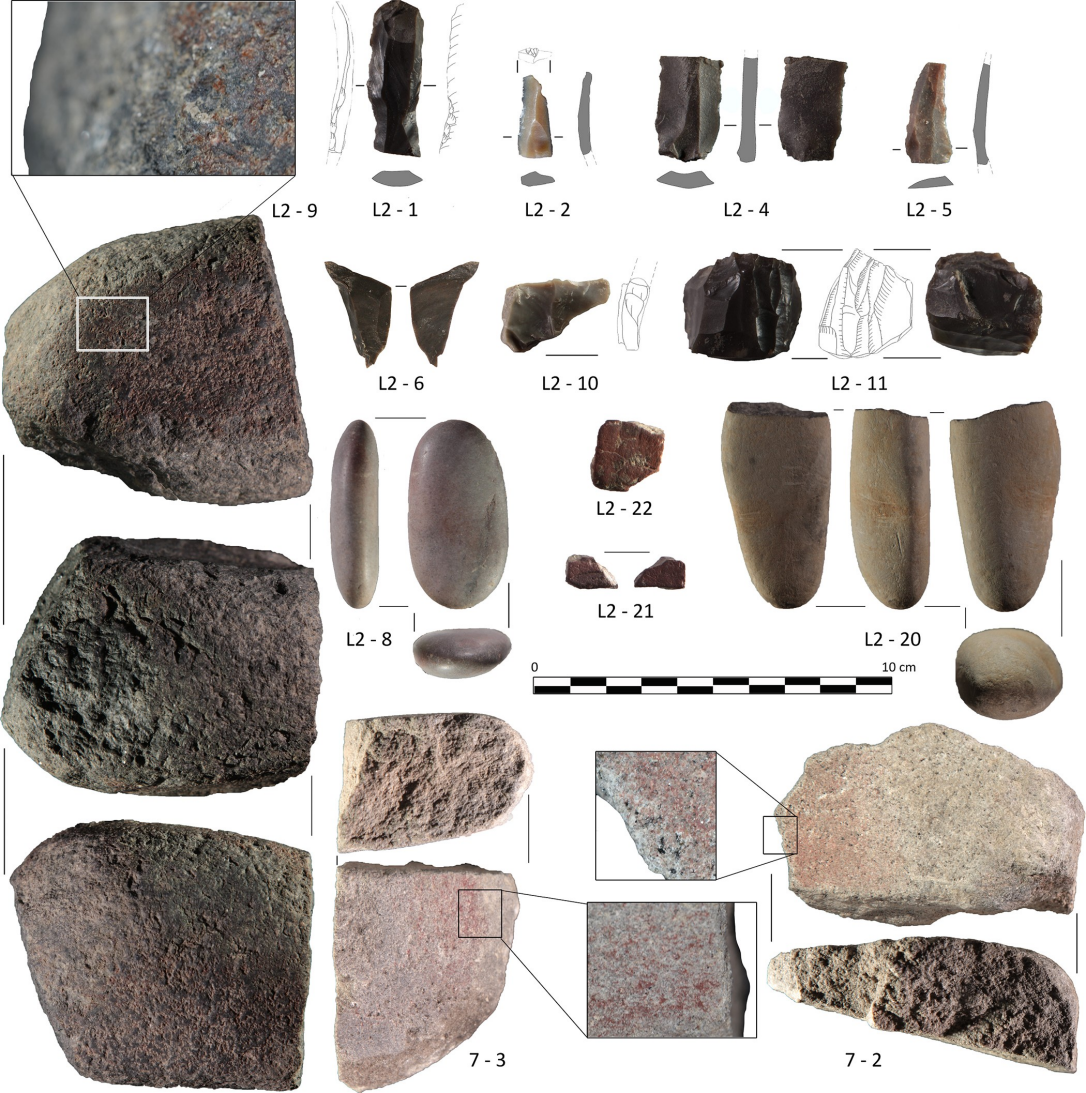
Selection of the lithic elements recovered from *Locus* 2.

A total of 31 stone tools have been documented (Table 6 and Fig 17), with an overall proportion of 45.16% of knapped lithic industry and 54.83% of polished stone. The former includes both the lithic products and the elements associated with their production, cores (14.29%) and reduction products (platform preparation and debitage, 7.14% each). The polished stone industry is made up of non-siliceous stone elements that have been used as tools for human activities and which are found in different degrees of processing.

**Table 6:** Lithic assemblage.

Units	Total	Typology				Nº	Retouched tool	%	Use-wear	%
7	3	Knapped 33.3%	Blade		100%	1	0	--	0	--
			Flake		0	0	0			
		Polished 66.7%	Quern		50%	1	--	--	2	100%
			Hand grinder		50%	1				
9	1	Knapped 100%	Blade		--	0	1	100%	1	100%
			Flake		100%	1				
*Locus* 2	24	Knapped 50%	Blade		41.7%	5	4	33.3%	2	16.66%
			Flake		33.3%	4	0			
			Core		16.7%	2	0			
			Debitage		8.3%	1	0			
		Polished 50%	Hand grinder		16.66%	2	--	--	9	75%
			Blade		16.66%	2				
			Polishing stone		16.66%	2				
			Indeterminate tool		16.66%	2				
			Ochre		16.66%	2				
			Unworked stone		16.66%	2				
Structure 1	3	Polished 100%	Stele	33.3%		1	--	--	1	33.3%
			Quern	33.3%		1				
			Unworked stone	33.3%		1				

With regard to the knapped lithic group, the lamellar forms stand out (42.86%) compared to flakes (28.57%) and other types -cores and debitage. The predominant flint is greyish (78.57%), followed by beige (14.29%), with only one case of brown flint. As for the heels, only 21.43% have preserved their entire proximal area, with three types of heels (smooth, dihedral and acute dihedral) with one case of each.

28.57% of the knapped lithic material has been retouched -all of them are lamellar forms-, with the predominance of simple marginal (33.3%) and abrupt deep modes in the same proportion, followed by one case of simple deep retouch and another of marginal plane. Direct orientation stands out with 66.67% of the cases. The predominant situation is bilateral, while continuous and denticulated delineations are also present. Among the cores, there is an equal representation of amorphous and carenoid cores. There are elements with tertiary extraction, as well as signs of thermal treatment.

The polished stone elements (Fig 17) include grinding tools (4 querns and 3 hand grinders), 2 polishing stones, 2 haematite nodules with abrasion traces, 2 elements of indeterminate function and the possible porous limestone stele of the stone platform. With regards to the raw materials, ophite, quartzite, sandstone and haematite are identified. Evidence of heating and polishing are found here in equal proportion (11.76%), only coinciding in one of the hand grinders. At the same time, there are traces of pigment on two of the querns, a hand grinder, and one of the polishing stones, representing 17.64% of the total. The grinding elements have flat, concave or convex surfaces, as well as traces of pitting from percussion. The polishing stone and the hand grinder with ochre remains were found precisely between the male skull and the sheep/goat remains.

**Fig 18:**
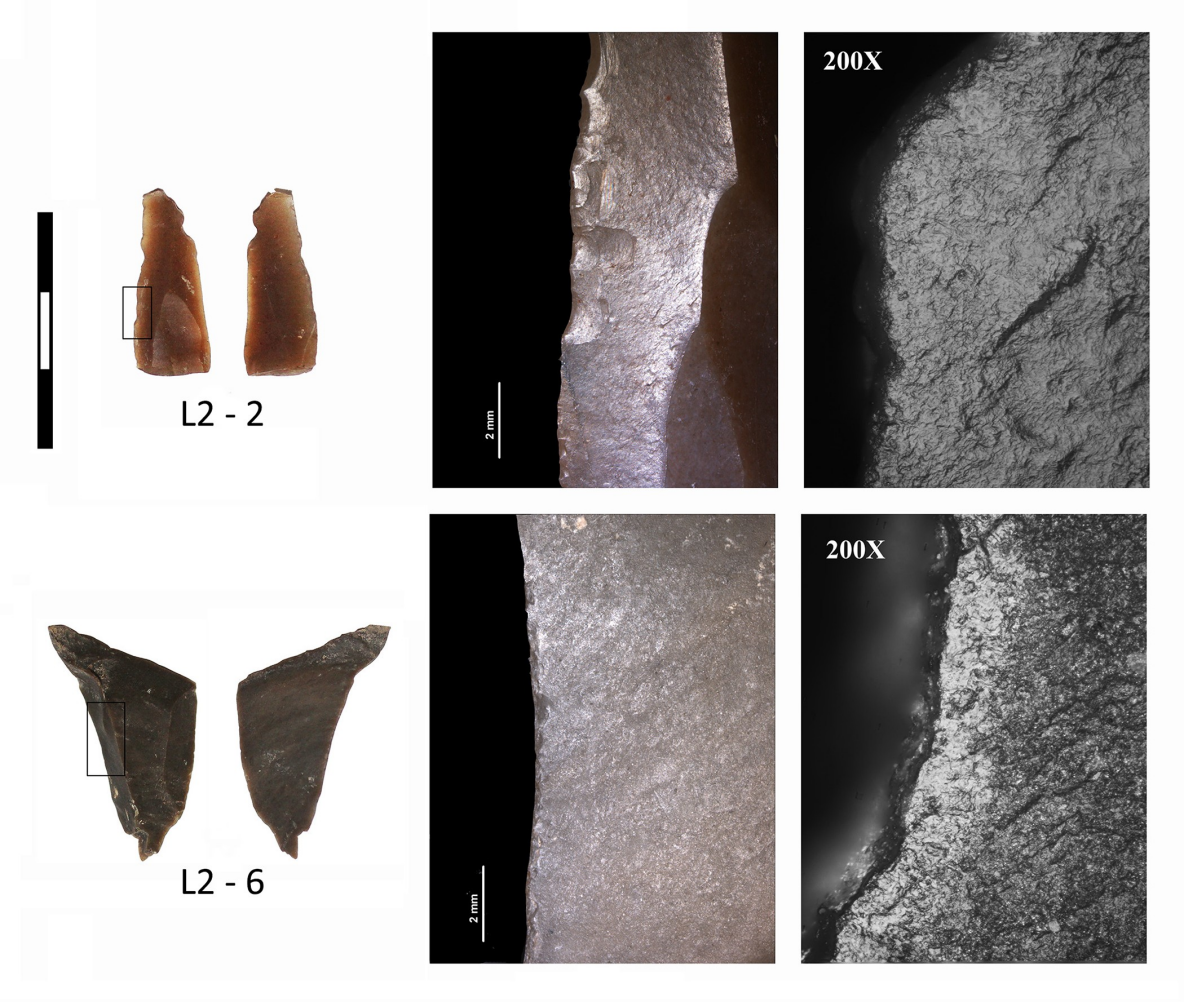
Examples of lithic elements with use wear evidence documented by traceological analysis: L2-2) distal fragment of a flint blade with retouch on both edges and evidence of dry skin scraping on the left edge; L2-6) flint flake probably used to scrape a mineral material.

A traceological analysis was carried out on a sample of 8 lithic elements, selected on the basis of visually identified use-wear and morphological characteristics in accord with potentially viable usage. It was possible to verify that 5 were used, 2 had no traces of use and 1 provided no criteria to determine whether it was used or not. In relation to the typology of the 5 used elements, 4 correspond to blades, 3 of which display retouch, and one is an unretouched flake. A diversity of uses and a maximum reuse of the functional capacities of these elements can be observed. This is illustrated by a distal fragment of a blade retouched on both edges. The left edge was used for scraping dry skin and the right edge for scraping dry skin and/or non-woody plants (Fig 18, L2-2). A proximal fragment of an unretouched blade (Fig 17, L2-4) was used to cut dry skin with one edge, and another half-distal fragment of blade retouched on both edges (Fig 17, L2-1), spatially associated with the sheep/goat, was used to scrape wood with one edge and wood or bone with the other. The fourth of the used blades is retouched on both edges, one of which was used to scrape a indeterminate semi-hard material (Fig 17, L2-5). Finally, a fractured flake (Fig 18, L2-6) shows use-wear linked to the scraping of a mineral material. The stone tools associated with this deposition may have been used to process dry skin, wood, mineral and perhaps bone.

#### 3.4.4. Plants

Seeds and wood preserved by charring have been recovered from the flotation of the sediments of these stratigraphic units. All of the identified seeds are cereal caryopsis, documented in the *Locus* 2 units and in the upper layer (Unit 7) of the hearth (Table 7). Here, 2 caryopsides of *Triticum sp*. and 4 indeterminate cereal fragments have been recovered. A slightly larger quantity appears in proximity to the skulls, including 1 caryopsis of naked wheat (*Triticum aestivum/durum*), 3 of *Triticum sp*. and 15 indeterminate cereal fragments.

**Table 7:** List of documented seeds.

Archaeological area	C006	
Units	7	*Locus* 2
Sample volume (L)	9	10
CULTIVATED PLANTS		
*Triticum aestivum/durum*		1
*Triticum* sp.		3
*Triticum* frag.	2	
Cereal frag.	4	15
WILD SPECIES		
Indet.	1	2
Total remains	1	4
No. of taxa	2	2
Density x 10 L	1,1	4,0

Given this small volume, it cannot be guaranteed that these remains formed part of the deliberate deposition of offerings. However, all of the taxonomically determined remains belong to wheat, among which only the presence of naked varieties has been confirmed. These data are consistent with the carpological record documented in contemporaneous levels documented in the sequence of another trench (named C003) located nearer to the entrance of the cave [89].

**Fig 19:**
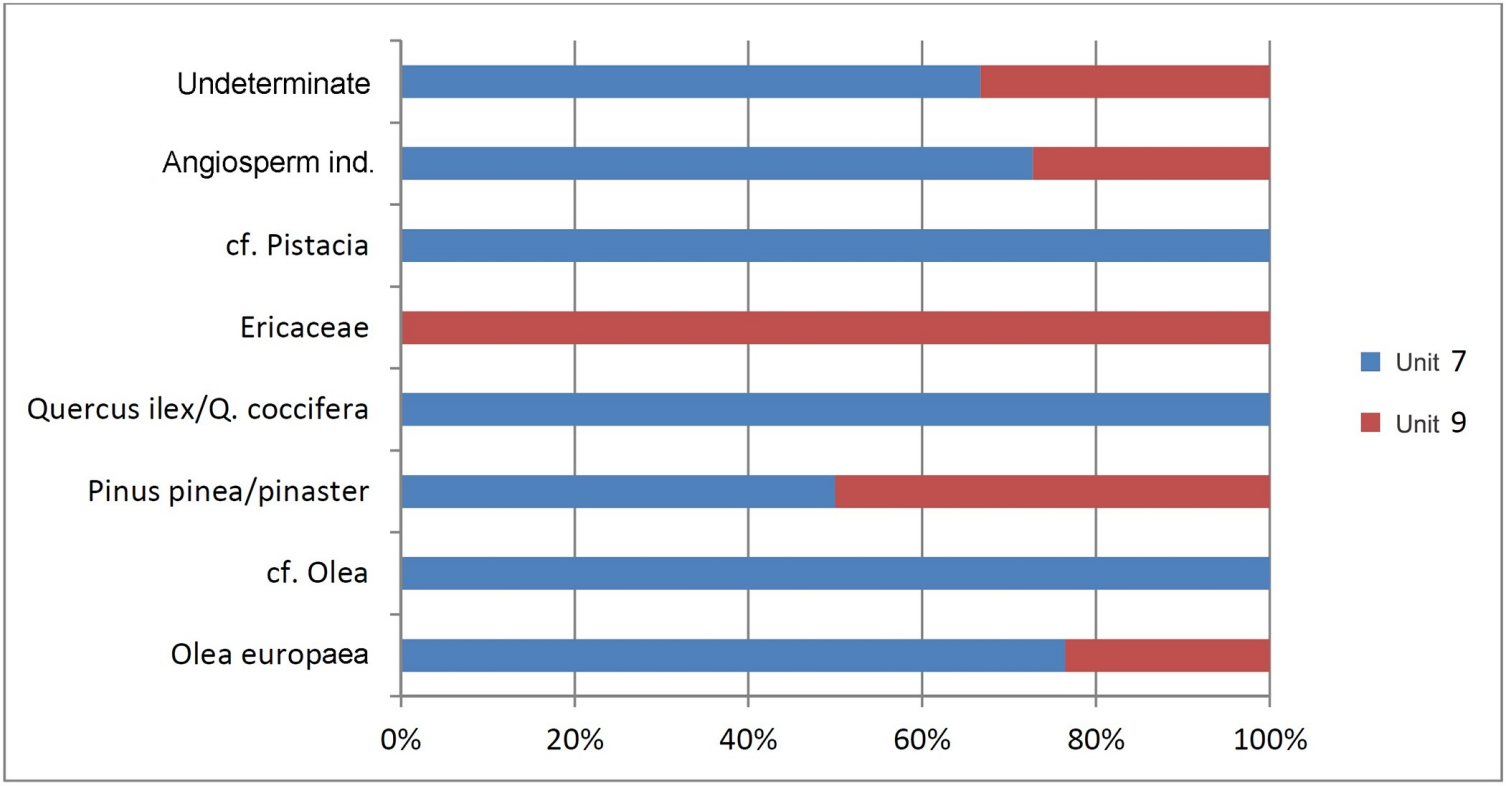
Histogram of the anthracological remains associated with hearth 7/9 of *Locus* 2.

Remains of charred wood have been documented mainly in the hearth made up of Units 7 and 9, and only occasionally in *Locus* 2. Those of the hearth can be assumed *in situ*, in a context where these plant resources were deliberately used and burnt (Fig 19, Table 8). A wide range of plant species, both tree and shrub, is documented. The remains of *Olea europaea* are predominant, followed by the indeterminate remains of *Angiosperm*, and occasionally *Pinus pinea/pinaster*, *Quercus ilex/coccifera*, *Ericaceae* and *Pistacia*. The predominance of the first two is corroborated by the results of the anthracological analysis carried out for the trench mentioned above located near the cave entrance (C003), in all the Neolithic levels containing charcoal remains. However, it is interesting to note that the pollen analysis carried out for the C003 trench showed significantly lower proportions of pollen from *Olea europaea* than from *Quercus ilex*, a taxon which is predominant in other contemporaneous levels [89].

**Table 8:** Charcoal remains from hearth 7/9.

	Unit 7	Unit 9	Total
*Olea europaea*	13	4	17
cf. *Olea*	2		2
*Pinus pinea/pinaster*	1	1	2
*Quercus ilex/Q*. *coccifera*	1		1
*Ericaceae*		1	1
cf. *Pistacia*	1		1
Angiosperm indet.	8	3	11
Indeterminate	2	1	3
TOTAL	28	10	38

This contrast between pollen and anthracological data may indicate a cultural pattern based on the differential use of *Olea europaea* with respect to other plant resources in this specific deposit. The concurrence of *Olea europaea*, *Quercus ilex/Q*. *coccifera* (oak) and *Pinus pinea/pinaster* (pine) is demonstrated in other Holocene sites studied in the northern regions of the Mediterranean basin [90]. However, *Olea europaea* remains the most used taxon at Dehesilla Cave, an observation that is consistent with the known exploitation of this wood as fuel [90].

### 3.5. Radiocarbon dates

From an initial series of 5, there are 3 successful C14 analyses: one from the sample from the right premolar 1 of Cranium 1 (the sample from Cranium 2 did not provide a date), another from the articulated sheep/goat skeleton and the last one from a fragment of *Olea europaea* charcoal from the hearth (a bone sample from this Unit did not provide a date either) (Table 9). These are AMS dates from analyses performed at the Centro Nacional de Aceleradores, University of Seville. The two former samples used Collagen extraction and Purification-Ultrafiltration method, while the latter used Acid-Base-Acid Cleaning method.

**Table 9:** Radiocarbon dates. Calibration with Calib 7.0 [91].

Lab Code	ID	Unit	Sample	%C	%N	C:N	BP Date	Cal BC—2 σ
CNA4494	DH17-22	*Locus* 2	*Homo*–Skull 1 Right PM1	37.8	14.6	3	5900±30	4840–4713
CNA4900	DH17-2	*Locus* 2	*Ovis aries/Capra* sp.–Femur	40.5	14.6	3.2	5870±30	4804–4683
CNA4485	DH17-3B	9	Charcoal–*Olea europaea*	—	—	—	5790±30	4713–4551

All three provide dates in the first half of the 5^th^ millennium cal BC, with some differences: 4840–4713 cal BC for the female skull, 4804–4683 for the sheep/goat and 4713–4551 for the hearth. The first two are very similar in date, thus it seems very likely that the death (and deposition) of the female individual–and possibly the deposition of the male individual (Cranium 2), given the stratigraphic association between the two- were contemporaneous to the death or sacrifice of the young sheep/goat. However, the date from Unit 9 of the hearth is slightly later. In order to explore the overall consistency of radiocarbon determinations, we carried out a chi-square statistic test [92] using the R_Combine parameter in Oxcal 4.3. The slightly later date provided by the hearth (5790±30) is the only one not supported statistically to 5%, df = 2, T = 7.164 (>5.991), but would be positive in goodness of fit for 0.975/α = 0.025 (<7.378). This implies several possible interpretative scenarios. We cannot rule out the possibility that either of the skulls came from a previous primary burial, specially Cranium 2, since it has no radiocarbon date that guarantees that it is contemporary to the young sheep or goat, as is the case for Cranium 1. In any case, it seems plausible and parsimonious that the set of elements was deposited at the same time. If so, the radiocarbon difference may be due to the different material natures of the samples themselves -two on bone and another on charcoal-, which have also undergone different chemical procedures, and the results may be due to a methodological bias. Another option would be that there may have been a time sequence in the formation of this group of deposits. If so, the skulls and the sheep/goat may have been deposited first, and the hearth created and used at a later date. In any case, on the basis of the detailed spatial stratigraphy, the hearth was produced at a time when the dividing wall between the hearth and *Locus* 2 already existed, since the remains of the hearth are abutted onto (and do not exceed) the wall. The sequence would therefore have had to be the deposition of the skulls/sheep/goat/wall construction, followed by the the hearth, involving a series of related ritual events over a period of time.

## 4. Discussion

### 4.1. Middle Neolithic society and funerary evidence at Dehesilla Cave

Radiocarbon dates and material culture, especially pottery, converge to indicate that the archaeological record of the context analysed in this paper is consistent with that of the period traditionally known as Middle Neolithic. The previous 2016 excavation season at Dehesilla Cave had already documented a Neolithic sequence in Trench C003 with a thick level, the materials and dates of which are similar to those presented here [19]. This layer, Unit 14, yielded a radiocarbon date around 4728–4549 cal BC from a sheep/goat molar, most probably an *Ovis aries*. This unit displayed a large assemblage of engraved pottery [19], which also characterises the context now treated. This decorative group is present in the most complete vessels described above, both in the context containing the skulls (*Locus* 2), as well as in the associated hearth (UE 7). This indicates not only a strong relationship (chronological, cultural and/or ethnic) between the possible sequence of events in the context now provided by C006, but also between this context and the population responsible for the material record found during 2016 in C003. This material record is attributed to the Middle Neolithic A in the proposed periodisation [19], while the Middle Neolithic B (spanning the second half of 5^th^ millennium BC) does not display this type of pottery. This defining pottery style, however, is only known in the south of the Iberian Peninsula, and with decorative compositions similar to those of Dehesilla Cave appears to be restricted to the caves of El Parralejo (San José del Valle) [93–95], Ánfora (Ardales) [96], Gato (Benaoján) [97] and the open air site of Esperilla (Espera) [98]. The initial multidisciplinary analyses on the Middle Neolithic A record of Trench C003 indicated populations with a strong agricultural component, centred fundamentally in the cultivation of naked wheat during the second quarter of the 5^th^ millennium cal BC [cf. 89].

Several elements appear to indicate the local character of the buried individuals. The cultivation of cereals requires sedentary or scarcely mobile populations throughout relatively reduced environments, since the developmental cycle of the crops takes place over a large part of the calendar year. The strontium isotope analyses that we have carried out for residential mobility are consistent with the local base-line. The limited distribution of the engraved pottery can also be considered. The maximum distance between the sites where this decorative style is currently documented is approximately 85 km. The isotopic values obtained for the two individuals sampled are consistent with the expected strontium sources for the limestone landscape of the outer Baetic mountain range, where the caves are located, as well as for the Neogene Basin where the open-air site is located. These individuals therefore probably belonged to rural populations with a sedentary behaviour and a relatively small range of mobility. It is impossible to establish whether they may have been direct descendants of other populations previously present in southern Iberia or not. Indeed, at Dehesilla Cave there is evidence that the populations corresponding to the Early Neolithic occupied this site during at least the second half of the 6^th^ millennium BC [19].

The excavations carried out at Dehesilla Cave during 1977 and 1981 documented a series of burials, although these mainly belong to the Early Neolithic (~8). Among these, there was an almost complete skeleton, belonging to a female individual based on the typically female traits of the pelvic bone and the gracile traits of the skull. Our two crania are dated in the Middle Neolithic, thus a direct comparison cannot be drawn. Also, from the morphological point a view, the two mesocephalic crania of *Locus* 2 are quite different from the dolichocephalic skull recovered in 1981. The only burial that was initially thought to belong to the Middle Neolithic [20] may, judging by the radiocarbon date obtained from an associated charcoal, be a Late Bronze Age/Early Iron Age burial [99]. Unit 14 in Trench C003 of the 2016 excavations, from the same period as the deposition analysed here, did not provide any funerary data, only two small indeterminate fragments from isolated adult human bones lacking any diagnostic traits.

The contrast in funerary use between the Early and Middle Neolithic periods of Dehesilla Cave is noteworthy, with around ten cases from the former period and, at present, only the new find presented in this paper from the latter. Moreover, this is not a common burial, such as a grave, but a complex depositional context located in the most inaccessible room of the cave and formed by two skulls without their jaws, surrounded by unusual stone structures and a hearth, and accompanied by animal offerings, artifacts and possible plant remains. This find is of great relevance, therefore, not only because of the scarce knowledge of the funerary practices of this period, but also because of its own singular characteristics.

### 4.2. The funerary record dated between 4800–4550 cal BC in the Iberian Peninsula

**Fig 20:**
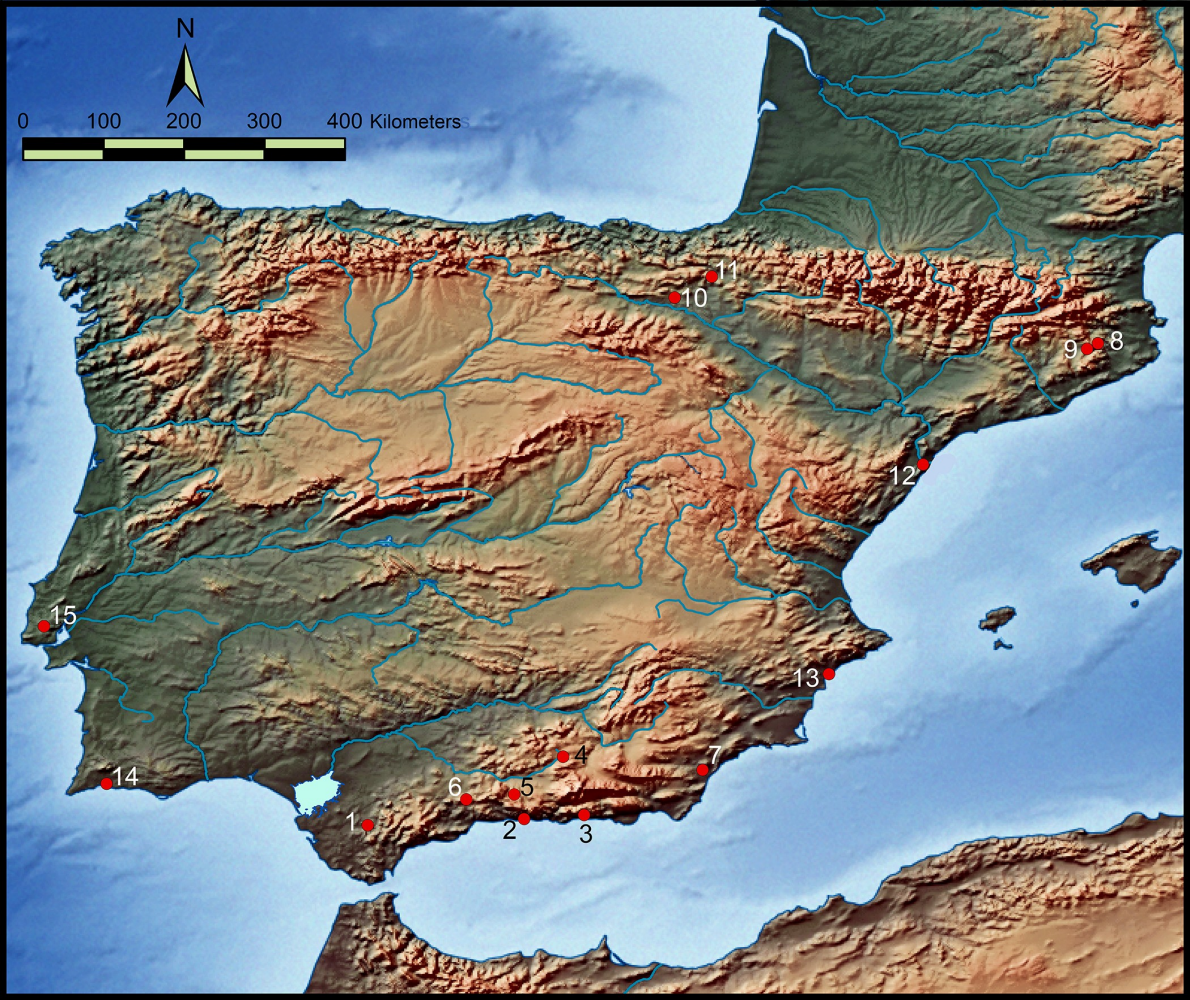
Location of the archaeological sites with funerary evidence for this period throughout the Iberian Peninsula: 1) Cueva de la Dehesilla; 2) Cueva de Nerja; 3) Cueva de los Murciélagos de Albuñol; 4) Cueva de la Carigüela; 5) Cueva del Agua; 6) Cueva de El Toro; 7) Cerro Virtud; 8) Cova d l’Avellaner; 9) El Padró; 10) Los Cascajos; 11) Paternanbidea; 12) Barranc d’en Fabra; 13) Tossal Basses; 14) Castelho Belinho; and 15) Salemas. (Theme map base: [122]. These sites are described in the same order in Table 11).

Table 10 contains only the known funerary sites with radiocarbon dates between 4800–4550 cal BC. The majority of burial evidence for this period in the Iberian Peninsula comes from primary burials, with only a few disarticulated or isolated remains documented (Fig 20 and Table 11). Secondary burials are rare, and the exclusive deposition of skulls is extraordinary. Only Cueva del Toro can be added to the case of Dehesilla Cave, although the former may belong to an earlier chronological phase corresponding to the traditional Andalusian Early Neolithic [109], that is to a period during which this type of deposition appears to have been more common. In the sample available and analysed here, with the exception of the frequent isolated remains and the secondary burials at Cueva de los Murciélagos and Cerro Virtud, the funerary spaces generally include several burials, in variable numbers, leading to their interpretation in some cases as collective burial areas, or even as true necropolis (Paternanbidea and Castelo Belinho).

**Table 10:** Available radiocarbon dates from archaeological sites with funerary evidence between 4800–4550 cal BC.

Site	Context	Sample	BP Date	CAL BC (2 sigma)	Lab Code	References
Cerro Virtud	Phase II	*Homo*	6030 ± 55	5200–4780	OxA-6714	[100]
	Phase II	Charcoal	5920 ± 70	4990–4610	Beta-90885	[100]
	Phase II	Charcoal	5895 ± 55	4910–4610	OxA-6715	[100]
	Phase II	Charcoal	5860 ± 70	4910–4540	Beta-101425	[100]
	Phase II	*Homo*	5840 ± 80	4900–4490	OxA-6580	[100]
	Phase II	Sediment	5830 ± 90	4910–4460	Beta-118938	[100]
	Phase II	*Homo*	5765 ± 55	4730–4460	OxA-6713	[100]
	Phase II	Charcoal	5660 ± 80	4690–4350	Beta-90884	[100]
Cueva del Toro	Phase IV	Charcoal	6030 ± 70	5210–4720	GrN-15444	[101]
	Phase IV	Homo	5980 ± 40	4988–4772	Beta-365292	[102]
	Phase IV	Charcoal	5820 ± 90	4900–4460	GrN-15440	[101]
Salemas		*Homo*	6020 ± 120	5230–4670	Icen-351	[103]
Paternanbidea	Burial 2	*Homo*	5960 ± 40	4942–4728	GrA-13675	[104]
Los Cascajos	Structure 196	*Homo*	5945 ± 95	5191–4555	Ua-24423	[104]
	Structure 265	*Ovis capra*	5640 ± 75	4660–4300	Ua-16025	[105]
Cova de l’Avellaner	Cav. Sepul. 3a	*Homo*	5830 ± 100	4935–4460	UBAR-109	[106]
	AV-CO I-X 25	*Homo*	5941 ± 34	4930–4725	CNA3304	[107]
Cueva de los Murciélagos, Albuñol		Esparto	5900 ± 38	4850–4680	CSIC-1134	[108]
		Esparto	5861 ± 48	4850–4590	CSIC-1132	[108]
Tossal de les Basses	Stone floor; UE34	Seed	5880 ± 50	4850–4600	Beta-232484	[109]
	Drainage ditch(4121) fill (149)	Seed		4720–4520	Beta-232483	[110]
	Burial 2–1819	*Homo*		4590–4450	Beta-225216	[110]
	Burial 9–11471	*Homo*		4590–4450	Beta-225223	[110]
Castelo Belinho	Structure 3	*Venerrupis*	5880 ± 55	4904–4598	Sac-2030	[111, 112]
	Structure 4	*Homo*	5720 ± 40	4685–4462	Beta-199913	[111, 112]
	Structure 53	*Homo*	5662 ± 32	4582–4374	Wk-28000	[111, 112]
Barranc d’ en Fabra	Post hole	Charcoal	5880 ± 110	5040–4510	BETA-61490	[113]
Cueva de Nerja	Vestíbulo. NE (1NE) Nerja-1-1829.	*Homo*	5875 ± 80	4940–4540	Ua-12467	[114–116]
	Torca. South Sect. NT 11e	Charcoal	5789 ± 40	4730–4530	Beta-168972	[116, 117]
	Torca. South Sect. NT-11c	Charcoal	5760 ± 40	4710–4500	Beta-195999	[116, 117]
	Sala del Cataclismo	Charcoal	5770±40	4717–4526	Beta-270018	[118]
		Charcoal	5760 ± 40	4710–4505	Beta-195998	[116]
		Charcoal	5740 ± 40	4696–4491	Beta-270037	[119]
El Padró II		Charcoal	5870 ± 100	4984–4499	UBAR-115	[120]
		Charcoal	5770 ± 80	4822–4451	UBAR-114	[120]
		Charcoal	5600 ± 130	4768–4076	UBAR-113	[120]
		Charcoal	5580 ± 130	4719–4070	UBAR-116	[120]
Cueva de la Carigüela	CIV 5, Level I-5		5690 ± 30	4610–4450	Pta-9162	[121]

**Table 11:** Ritual features of funerary sites throughout the Iberian Peninsula.

Site	Site Type	Human bones	Burial structures	Associated structures	Position	Orientation	Pathology	Offerings / material culture	References
	Access difficulty								
Cueva Dehesilla	Cave	Crania without mandibles	Area delimited by stone blocks and wall	Hearth and stone platform		1: Skull to S facing E;	Incomplete trepanation; cut mark on the occipital bone	Young sheep/goat, 4 pots, lithics, Triticum, *Olea europea*, *Pinus pinea/pinaster*, *Quercus ilex/coccifera*, *Ericaceae* and *Pistacia*	[21]
	Difficult					2: Skull to N facing W			
Cueva Nerja	Cave	Individual burial and relocated remains	Simple pit	None	Dorsal decubitus?	NW-SE	Dental wear	*Iberus alonensis*, limestone object	[114, 115]
	Easy								
Cueva Murciélagos	Cave	Primary and secondary burials	Communal room	—	—	—	—	Basketry, *Papaver somniferum*	[108, 123]
	—								
Cueva Carigüela	Cave	Individual burials and relocated remains	—	Hearths (habitat?)	—	—	Evidence of flesh removal?	Schist, limestone and marble bracelets, beads?	[7, 123–125]
	Intermediate								
Cueva Agua	Cave	Individual burials	Pits marked by stone blocks	Hearth (both)	Lateral decubitus (both)	—	—	Undecorated bowl	[126, 127]
	—							Flint blades, fossil corral, limestone bracelet?	
Cueva Toro	Cave	Skull and other parts (secondary burial?)	None	—	—	—	Incisions (flesh removal)	Ochre	[101, 128, 129]
	Difficult							Two undecorated bowls and the base of a third	
Cerro Virtud	Open air	Primary and secondary burials and isolated remains	Collective pit	Hearth, delimitation stones (wall)	Left lateral decubitus	W; E; N or S?	—	Undecorated pots and bowls, flint lithics, beads (stone and shell), perforated *Columbella rustica*	[100, 130]
Cova Avellaner	Shelter	Disarticulated remains	None	Separation walls	—	—	Caries, periodontitis and arthritis	Montboló style pottery, lithics, spatulas and other bone tools, bracelets, beads, perforated wild boar tusks, *Cardium edule* shells and faunal remains	[3, 107]
El Padró	Open air	Individual burial	Grave	—	Flexed (lateral decubitus?)	—	—	Motboló style geometric pottery, arrowheads	[3, 15, 131]
Los Cascajos	Open air	Individual burial	Pit	Habitat structures and hearths	Flexed (lateral decubitus?)	—	—	Bowl and other impressed and sgraffito pottery, bone tools and horn awls, hand grinders	[104, 105, 132]
Paternanbidea	Open air	Individual burials	Pit	Habitat structures	—	NE-SW, heads to the E-NE	—	Impressed bowl, geometric microliths, beads and bracelet	[104, 133]
Barranc Fabra	Open air	Powdered remains	Vertical stone slab structures	Habitat structures	—	—	—	Pottery bottle, lithics, bracelets and shell (*Pecten*, *Cardium*) and limestone beads	[3, 113, 134]
Tossal Basses	Open air	Individual burials	Pits	Habitat structures	Lateral decubitus	N-S; Head to the W; NW	—	Tomb 14: three complete pots, characteristic of the postcardial or comb decorated horizon	[110, 135]
Castelo Belinho	Open air	Individual burials	Pits	Habitat structures	Lateral decubitus	—	Caries	Bowls and globular pots, *Glycymeris bimaculata* bracelets, flint flakes and blade, hammers and querns, ochre and fauna	[112]
Salemas	Open air	Individual burials	Karst depressions	Habitat structures	—	—	—	Boquique style impressed pottery	[136]

The type of funerary structure characteristic of the known burials is usually the individual grave, in some cases delimited by stone blocks, although there are examples where the burials were placed directly on the ground. In caves, the information available on the spatial location of burials is sparse. At least at Dehesilla Cave and perhaps Cueva de la Carigüela, areas of difficult access appear to have been preferred, as they are the furthest away from the entrance to the cave and/or because they constitute geomorphologically inaccessible spaces. However, an opposite example is provided by the location of burials at Cueva de Nerja.

In a large part of the comparative sites, the burial contexts usually display combustion structures or hearths. There are hardly any known cases of other associated structures in caves. Dehesilla Cave has a delimitation or boundary wall between the deposition of the skulls and the rest of the associated structures: the aforementioned hearth and a stone platform. Cova de l'Avellaner also has walls creating the spatial differentiation between the three cavities of the shelter. And at Cueva del Agua, one of the burial pits displayed a stone paving, cover and perimeter ring. In the case of funerary contexts located in the open, there is a delimiting wall between a hearth and the deposition of the bodies in the collective grave of Cerro Virtud. At open-air sites that also display habitat contexts, there are not only hearths, but also numerous types of pits and negative features, as well as huts of different shapes.

With the exception of Nerja, the bodies are usually placed in a lateral, more or less flexed position. The orientation of the burials is extremely varied, and does not appear to show significant differential patterns. Paleopathological analyses are scarce and probably unequal in different osteological assemblages. In relation to the skulls from Dehesilla Cave (one showing possible trepanation and cut mark), the possible evidence of defleshing from Cueva de la Carigüela is worthy of special mention.

A similar observation applies to the availability of information regarding funerary offerings. The reliability of data regarding burial goods is even more uneven between sites, especially since some of it comes from relatively old excavations. However, several aspects can be noted. Animal offerings are not common. Apart from Dehesilla Cave, only a very few cases are known: Cova de l'Avellaner with sheep/goat and deer remains, and Castelo Belinho, as well as the indeterminate remains of ritual pit nº 256 at Los Cascajos -without human remains but perhaps associated with other burial pits. Shells (malacofauna) are more common, having been documented at Cueva de Nerja, Cerro Virtud, Cova de l'Avellaner, Barranc d'en Fabra and Castelo Belinho. Most of the analysed deposits contain pottery. In many of them, they are entirely or essentially plain pots -for instance at Cueva del Agua, Cueva del Toro, Cerro Virtud, Cova de l'Avellaner, El Padró, Los Cascajos and Tossal Basses. Two of the four vessels directly associated with the deposit at Dehesilla Cave are undecorated. There is evidence of knapped lithic industry in one third of the funerary contexts analysed, and regarding polished stone industry the fragments of grinding stones and the possible use of ochre at Castelo de Belinho provide similar evidence to that documented at Dehesilla Cave. In *Locus* 2, the entire bone tool repertoire is limited to one spatula made from a deer metatarsus. At the rest of the comparative sites, bone industry is minority, if not altogether absent, with the exceptions of Los Cascajos and the extraordinary case of Cova de l'Avellaner, with a numerous and diverse bone assemblage. The results of possible carpological and anthracological analyses in the contexts analysed here have not generally been made known, thus no comparisons can be made.

### 4.3. Interpretative possibilities of the *Locus* 2 deposition

The *Locus* 2 depositional context at Dehesilla Cave displays several characteristics that are different from those of the rest of the known funerary contexts of the 4800–4550 cal BC period in the Iberian Peninsula. The exclusive selection and (re)burial of the skulls is contrary to its interpretation as a primary burial. It is the only case dated with certainty to the Middle Neolithic that indicates a selective treatment and burial of human skulls (without the mandible). It also provides a novel archaeological context with several structures as peculiar as the stone platform described above (Structure 1).

These exceptional characteristics may correspond to the hypothesis of a deposit (and activities) with a strong ritual component, beyond that which is usually shown by the archaeological record related to the common (or normative) burials of these populations. The very location of the deposition in one of the areas furthest from the cave entrance, in a sort of hidden and secluded chamber, is suggestive in this sense.

Deciphering these possible ritual activities is clearly no simple task, but at least the possible lines of interpretation of this record can be tentatively pointed out. The possibility of being a deposit of isolated individuals, as a kind of funerary ostracism, does not seem to be in line with the investment in the creation of the documented stone structures. The *ex novo* purpose-specific construction of these structures is also not usual in normal Neolithic secondary burials. Based on the durable elements of the archaeological record, the practices involved in secondary burials are usually limited to the selection of all or only part of the remains to be moved between locations, but without the association of structures such as those documented in this case and without additional rituals such as the sacrifice and *in situ* deposit of the infantile sheep/goat. Although the possibility that either of the skulls (or at least Cranium 2) could have come from a primary burial prior to deposition cannot be completely ruled out, it must be emphasised that, among the funerary contexts dated in the same chronological period throughout the Iberian Peninsula, the practice of primary burial is predominant, and secondary burial is only rarely documented (if at all at the Cueva de los Murciélagos and Cerro Virtud). In any case, the characteristics of this deposit indicate some type of singular and intentional social treatment by the community through the enactment of a special deposition with a strong ritual component. Along these lines, the deposit may indicate some type of honorary recognition, related to social leaders, perhaps great figures or religious guides.

The presence of the cut mark on the occipital of Cranium 1 was performed post-mortem, but before the loss of muscle tissue, and therefore hypothetically as a means of separation of the skull or decapitation of the individual. Although it cannot be stated categorically, it is quite possible that death was chronologically close to the time of deposition of the skull (if not at the time of death itself, before complete tissue decomposition). This same (female) individual had also previously suffered a probable incomplete trepanation, during life, since the bone regeneration observed indicates that this happened years before her death. This previous event may therefore have no direct relation to the events and ritual now examined. On the male skull (Cranium 2) no cut marks or evidence of this kind has been observed. However, the stratigraphic information guarantees the contemporaneous deposition of both skulls and several other elements of the context. Although the simultaneous natural death of both individuals (or the above mentioned secondary burial of one of them) cannot be ruled out, the natural death one and the ritual sacrifice of the other, or the sacrifice of both, may be equally likely.

The empirical data thus opens up new anthropological scenarios, perhaps sacrifices (human and animal) related to propitiatory activities, divine prayers and/or commemorative festivities (cosmogonic, seasonal rites…). In this sense, it is necessary to recall the possible seasonal character of the deposition in Spring (based on the plausible sacrifice of the young sheep/goat) and, indeed, to highlight the stone platform, located within a natural niche in the cave wall, which may have functioned as a kind of altar, and also the decorative features of the two engraved pottery vessels, one of them physically associated to the body of the young sheep/goat and the other found in the hearth. The latter, in fact, displays a unique composition, a possible dynamic schematic scenary formed by a series of vertical branching motifs that occupy almost the entire height of the vessel. The repetitive motif appears sometimes in other Neolithic pottery cases [137–139] but also recalls certain vegetal motifs of Iberian rock art (named ‘ramiform’ or branch-shaped [140]), and indeed is common in caves and shelters throughout the region [see for example 141–144]. Therefore, the relationship previously suggested by other authors between Neolithic pottery decoration and schematic cave art [108, 145] is certainly a line to follow up in future. Some of the possibilities mentioned above, especially those that indicate the possibility of a temporal sequence in the completion of the depositional context, would imply a succession of ritual events in accordance with one or several of these interpretative scenarios, in which some kind of ancestral cult could also be present.

## 5. Conclusions

Given the relatively diverse panorama, it is worth asking if there was in fact a cultural homogeneity behind the peninsular funerary record between 4800–4550 cal BC. It is interesting that Andalusian funerary evidence for this period comes from caves, with the exception of the anomalous case of Cerro Virtud. Meanwhile, in the rest of the Iberian Peninsula where funerary evidence from this chronological period exists -that is, fundamentally in the eastern half of the peninsula, and the occasional case in South and Central Portugal- it comes instead from open-air sites (with the exception of the Cova de l'Avellaner shelter). It is unlikely that this dichotomy could be due solely to methodological biases in the surveying and search for prehistoric sites in the different regions. Moreover, most of these regions also have shelters and caves, so the differential availability of suitable sites cannot be the only explanatory factor either. It is therefore possible to think of a cultural mosaic in the funerary traditions of the Iberian Peninsula during these centuries of the first half of the 5^th^ millennium cal BC, leading to the observed dichotomy between the Andalusian region and, particularly, the eastern half of the peninsula. In the former, similarly to the southern regions of Portugal, open-air burials only appeared towards the end of the 5^th^ and especially during the 4^th^ millennium cal BC, many of them associated with the emergence of ditched enclosures and megalithic monuments.

Despite this possible caveat regarding the heterogeneity of the funerary rites of this chronological interval in the Iberian Peninsula, taken as a whole it is possible that there may be a greater similarity between the practices of the early Middle Neolithic and the funerary customs of the previous Early Neolithic than between the former and those of the period after the mid-5^th^ millennium cal BC. This point of discussion requires a systematic comparative approach to the overall body of Neolithic funerary evidence, which greatly exceeds the scope of the present work. However, the basis of the hypothesis may be disclosed as the observation that the bulk of the funerary evidence is found within habitat areas, either in caves or outdoors. Only in the cases of Paternanbidea and Castelo Belinho the burials form a more or less specific funerary area, a kind of necropolis. These, however, already display some of the characteristic features of later sites, some of them with numerous excavated features, small circular pits and large enclosures, and/or megalithic constructions. Close to Dehesilla Cave, for example, representative sites of the funerary rites of the end of the 5^th^ and 4^th^ millennia cal BC are the settlements of Campo de Hockey [146] and the megalithic necropolis of Cañada Real and El Palomar, at Los Molares [147], or the megalithic necropolis of Alberite [148]. More or less contemporaneous is the well-known horizon of the pit fields of the northeast of the peninsula.

This possible greater cultural distance between the records of the two periods may be at least partially due to the alleged population decline during the traditional Middle Neolithic period, although this general demographic dynamic surely had great differences and regional rhythms throughout the Iberian Peninsula. This panorama can be generally extrapolated to the best-part of the Iberian Peninsula. The number of radiocarbon dates available for the two central quarters of the 5^th^ millennium cal BC is notably lower than for the previous and subsequent periods (Early and Late Neolithic, respectively) [cf. 149]. In the south of the Iberian Peninsula, the site of Los Castillejos, in Las Peñas de los Gitanos (Montefrío, Granada), is of particular relevance in this regard, due to the intense program of archaeological activities carried out, and where a period of abandonment of the site has been documented between ca. 4800-4400/4200 cal BC [150]. In fact, this specific period has very few chronological parallels in Andalusian sites that are well dated from domestic and/or short-lived elements, although there are some scarce dates that cover the second and third quarters of the 5^th^ millennium BC from a scatter of cave and open-air sites in the Mediterranean coastal regions and southeast Andalusia. If there were a similar hiatus at Dehesilla Cave, however, it may only have been a little later, from the middle of the 5^th^ millennium cal BC, in the period known as Middle Neolithic B, where it is noted, for example, that the volume of archaeological material remains decrease sharply with respect to the previous period analysed in this work [19].

If this hypothesis were true, the lower number and density of populations during this period might have caused a greater isolation of the groups from one another, and therefore led to a lower rate of interaction and horizontal transmission than those of the Early Neolithic and, of course, of the Late Neolithic, when numerous new features are differentially replicated and transmitted rapidly between populations, such as the emergence of pit enclosures and the consolidation of necropoli, as well as megalithism from the beginning of the 4^th^ millennium cal BC.

At any rate, *Locus* 2 of Dehesilla Cave constitutes an extraordinary find and an important contribution to the current funerary evidence available in the Iberian Peninsula for the 5^th^ millennium BC. It has provided a great wealth of data that converges towards the ritual nature of this deposition. Moreover, its discovery opens up new possibilities for understanding the symbolic and the ritual social behaviours of the Neolithic agricultural populations of the western regions of the Mediterranean.
